# Exploring the therapeutic potential of Thai medicinal plants: in vitro screening and in silico docking of phytoconstituents for novel anti-SARS-CoV-2 agents

**DOI:** 10.1186/s12906-024-04586-z

**Published:** 2024-07-19

**Authors:** Bussayarat Maikhunthod, Sukanya Chaipayang, Akanitt Jittmittraphap, Narin Thippornchai, Pakpoom Boonchuen, Panlada Tittabutr, Griangsak Eumkeb, Sahachai Sabuakham, Thanyada Rungrotmongkol, Panupong Mahalapbutr, Pornsawan Leaungwutiwong, Neung Teaumroong, Waraporn Tanthanuch

**Affiliations:** 1 School of Biotechnology, Institute of Agricultural Technology, Suranaree University of Technology, Nakhon Ratchasima, 30000 Thailand; 2 Synchrotron Light Research Institute (Public Organization), Nakhon Ratchasima, 30000 Thailand; 3 Department of Microbiology and Immunology, Faculty of Tropical Medicine, Mahidol University, Bangkok, 10400 Thailand; 4 School of Preclinical Sciences, Institute of Science, Suranaree University of Technology, Nakhon Ratchasima, 30000 Thailand; 5 Department of Biochemistry, Center for Translational Medicine, Faculty of Medicine, Khon Kaen University, Khon Kaen, 40002 Thailand; 6 Program in Bioinformatics and Computational Biology, Graduate School, Chulalongkorn University, Bangkok, 10330 Thailand; 7 Center of Excellence in Structural and Computational Biology, Department of Biochemistry, Faculty of Science, Chulalongkorn University, Bangkok, 10330 Thailand

**Keywords:** Thai medicinal plants, Anti-SARS-CoV-2, COVID-19, LC–HRMS, Molecular docking, Mulberry, Flavonoids

## Abstract

**Background:**

The high virulence of severe acute respiratory syndrome coronavirus 2 (SARS-CoV-2), responsible for coronavirus disease 2019 (COVID-19), has triggered global health and economic concerns. The absence of specific antiviral treatments and the side effects of repurposed drugs present persistent challenges. This study explored a promising antiviral herbal extract against SARS-CoV-2 from selected Thai medicinal plants based on in vitro efficacy and evaluated its antiviral lead compounds by molecular docking.

**Methods:**

Twenty-two different ethanolic-aqueous crude extracts (CEs) were rapidly screened for their potential activity against porcine epidemic diarrhea virus (PEDV) as a surrogate using a plaque reduction assay. Extracts achieving ≥ 70% anti-PEDV efficacy proceeded to the anti-SARS-CoV-2 activity test using a 50% tissue culture infectious dose method in Vero E6 cells. Molnupiravir and extract-free media served as positive and negative controls, respectively. Potent CEs underwent water/ethyl acetate fractionation to enhance antiviral efficacy, and the fractions were tested for anti-SARS-CoV-2 performance. The fraction with the highest antiviral potency was identified using liquid chromatography–high-resolution mass spectrometry (LC–HRMS). Molecular docking analyses of these compounds against the main protease (M^pro^) of SARS-CoV-2 (6LU7) were performed to identify antiviral lead molecules. The top three hits were further evaluated for their conformational stability in the docked complex using molecular dynamics (MD) simulation.

**Results:**

The water fraction of mulberry (*Morus alba* Linn.) leaf CE (WF-MLCE) exhibited the most potent anti-SARS-CoV-2 efficacy with low cytotoxicity profile (CC_50_ of ~ 0.7 mg/mL), achieving 99.92% in pre-entry mode and 99.88% in postinfection treatment mode at 0.25 mg/mL. Flavonoids and conjugates were the predominant compounds identified in WF-MLCE. Molecular docking scores of several flavonoids against SARS-CoV-2 M^pro^ demonstrated their superior antiviral potency compared to molnupiravir. Remarkably, myricetin-3-*O*-β-D-galactopyranoside, maragrol B, and quercetin 3-*O*-robinobioside exhibited binding energies of ~  − 9 kcal/mol. The stability of each ligand–protein complex of these compounds with the M^pro^ system showed stability during MD simulation. These three molecules were pronounced as antiviral leads of WF-MLCE. Given the low cytotoxicity and high antiviral potency of WF-MLCE, it holds promise as a candidate for future therapeutic development for COVID-19 treatment, especially considering its economic and pharmacological advantages.

**Supplementary Information:**

The online version contains supplementary material available at 10.1186/s12906-024-04586-z.

## Background

A novel coronavirus disease 2019 (COVID-19) is an infectious viral illness caused by severe acute respiratory syndrome coronavirus 2 (SARS-CoV-2). The global outbreak of COVID-19 has caused rapid person-to-person transmission and high mortality rates [1]. Battling with SARS-CoV-2 infection could affect all groups of people, regardless of age and gender. COVID-19 not only affects human health, but also significantly affects mentality. Almost 60% of the university students in Bangladesh exert an extreme fear of academic delay and suffering from severe psychological stress from online education during COVID-19 pandemic [2].

Despite the rapid development of vaccines, they do not provide a 100% guarantee of efficacy, and reinfections can even occur in the previously infected individuals [3, 4]. The reasons may be the mutations in the virus itself and the host-immune responsive characteristics. The SARS-CoV-2 Delta variant (B.1.617.2) causes extremely more serious symptoms compared to the alpha strain. The variant showed high resistance to vaccines, resulting in breakthrough infections and an increased risk of reinfection. Omicron variant also proved to invade the vaccine-induced immunity of individuals resulting in the reinfections [5]. The limited of vaccine efficacy was suggested that the generated antibodies might not be able to recognize the dominant epitopes on the spike protein of the variants [4, 5]. Thus, a vaccine that is capable of inducing a robust immune response as well as other medical treatments should be further pursued.

Several antiviral medications have been repurposed, such as favipiravir, remdesivir, and molnupiravir. However, their use has been associated with various side effects, especially people in vulnerable groups [6–8]. A pregnant woman is considered highly susceptible to the SARS-CoV-2 virus due to the changes in her immunological, respiratory, and cardiovascular systems during pregnancy. These alterations have caused difficulty in therapy once the disease has developed [9, 10]. Although some drugs, such as chloroquine, established the safety of use for fetuses and expecting mothers [11], an elevated dose also has an adverse effect [9]. Moreover, the irregulated immune responses cause extensive systematic damage due to the overproduction of cytokines (cytokine storm) and chemokines from the hyperactivation of inflammatory and immune responses after infection [10]. Cytokine storm could lead to multi-organ failure and affected the long COVID symptoms and the subsequent chronic infection [12, 13]. Thus, these points should be considered in drugs development.

An alternative use of medicinal plants which contain a wide range of secondary metabolites possess several pharmacological activities, such as immunomodulatory, antibacterial, antiviral, antioxidant, and anti-inflammatory effects, to support COVID-19 patient treatment. Application of traditional Chinese medicine (TCM) as supportive agents with modern medicines were proposed as potential treatments to control the elevated cytokine storm [12]. Many research groups have shown a particular interest in exploring plants and their derivatives as potential natural candidates for combating SARS-CoV-2 [14–29]. Tallei et al. (2021) proposed green tea produced from *Camellia sinensis* (L.) contains (-)-epigallocatechin-3-gallate (EGCG), exhibited immunomodulatory, antibacterial, antioxidant, and anti-inflammatory effects. Besides, eight polyphenolics from green tea leaves extract were found to be able to in silico interact with main protease of SARS-CoV-2 (M^pro^). It reveals the potential use of medicinal plant extract in the management of COVID-19 with vaccination [28].

In Thailand, *Andrographis paniculata* (AP) which contains andrographolide, gained significant attention due to its extensive use in traditional medicine as a remedy for common ailments such as the common cold, diarrhea, and fever, and was initially recommended for use in the treatment of mild COVID-19 cases. Investigation the anti-SARS-CoV-2 activities of AP extract and andrographolide compounds in human lung epithelial cells and their cytotoxicity in major organ cell lines indicated a significant inhibitory effect on viral replication within acceptable ranges of cytotoxicity, thus revealed a possible post-infection treatment [20]. The clinical trial with mild COVID-19 patients using a daily 180 mg dose of andrographolide for 5 days during early treatment indicated promising efficacy of AP, including a more effective pneumonia prevention rate as observed through chest radiography [30]. However, a cohort study found contrasting results. AP-treated patient group exhibited a higher percentage of pneumonia cases than the control group, although the difference was not statistically significant [31]. Another Thai medicinal plant, *Boesenbergia rotunda* (BR), the BR extract and its phytochemical compound, panduratin A, possess potent in vitro anti-SARS-CoV-2 activity in both the previral entry and postinfection phases, with the ability to suppress viral infectivity in human airway epithelial cells. Clinical trials assessing the efficacy and safety of BR extract are ongoing [16]. *Artemisia annua* L. has also garnered researchers’ attention due to its long-term use in fever treatment in Southeast Asia and for patients experiencing respiratory distress. Several *Artemisia* extracts effectively inhibited SARS-CoV-2 and feline coronavirus infections [23]. However, the ability of *Artemisia* extracts to inhibit SARS-CoV-2 infection is not solely attributed to their major component, “artemisinin”. Instead, it is likely due to a combination of components working to block viral infection at the step of entry [18]. Despite extensive global research into natural extracts that are effective against SARS-CoV-2, several have emerged as intriguing candidates [16, 18, 20–24]. To the best of our knowledge, the clinical evidence of such candidates, both in terms of safety and effectiveness specific to COVID-19 therapy, including the study of their insight into antiviral mechanisms, remains restricted. Thus, there is still an opportunity to investigate further natural extracts or compounds to tackle this novel coronavirus.

In this study, we aimed to explore more potential Thai herbs as candidates against SARS-CoV-2 since Thailand is one of the countries rich in medicinal plants and herbal medicine recipes. Nineteen Thai medicinal plants, known for their antiviral properties or traditional use in fever or flu remedies, were investigated. Their ethanolic-aqueous crude extracts (CEs) were screened through the rational study design, mainly via in vitro antiviral activity assays. Furthermore, the promising extract’s phytochemical contents were revealed, and the prediction of their abilities to interact with the target viral protein, M^pro^ of SARS-CoV-2, through in silico molecular docking analysis and molecular dynamics (MD) simulation was also engaged.

### Study design

Figure S1 illustrates an overview of the study design (Additional File 1). Leveraging the containment capabilities of biosafety level 3 (BSL3) facilities, the antiviral efficacies of the obtained ethanolic-aqueous CEs from the 19 selected plants were preliminarily assessed against porcine epidemic diarrhea virus (PEDV) using a plaque reduction assay. Previous studies have indicated that using PEDV as a surrogate provides reliable results since SARS-CoV-2 and PEDV belong to the genus Alphacoronavirus within the Coronaviridae family, sharing similar structural characteristics [32–35]. CEs with ≥ 70% anti-PEDV efficacy were tested for their anti-SARS-CoV-2 activity in Vero E6 cells using the 50% tissue culture infectious dose (TCID_50_) method, based on pre-entry and postinfection modes and cytotoxicity evaluations.

To enhance antiviral efficacy, the potential anti-SARS-CoV-2 CEs underwent water/ethyl acetate fractionation to obtain their water fractions (WFs) and ethyl acetate fractions (EFs), which were then tested for anti-SARS-CoV-2 activities. The fraction displaying the most outstanding antiviral efficacy was identified for its tentative chemical content using liquid chromatography–high-resolution mass spectrometry (LC–HRMS). Notably, column chromatography (CC) was employed to partially separate the chosen WF or EF into several fractions before LC–HRMS to oversimplify the complexity of LC column separation. This contributed to more accurate compound identification. The tentatively identified compounds were evaluated for their interaction with the SARS-CoV-2 M^pro^ through in silico molecular docking, indicating potential antiviral lead compounds. The top three hits were analyzed to determine their structural stabilities in the ligand–protein interaction through all–atom MD simulation.

## Material and methods

### Plant materials

In this study, 19 medicinal plants were selected from common Thai herbs gathered from farms in Thailand. Taxonomic identifications were conducted by Asst. Prof. Dr. Santi Watthana, affiliated with the School of Biology at the Institute of Science, Suranaree University of Technology, Thailand. Voucher specimens of these plants were deposited at the Thai Traditional Medicine Herbarium in Thailand. The assigned specimen numbers, plant parts used for extraction, and harvesting areas are provided in Table S1 (Additional File 1). Twenty-two different plant parts were examined to account for varying key phytochemicals distributed in various parts of plants. The selected herb parts were initially shredded, oven-dried at 40°C, and subsequently ground into a powder.

### Preparation of plant extracts

#### Preparation of crude extract

The experiment was conducted following previous studies with slight modifications [36, 37]. The plant powder was extracted by maceration in 80% ethanolic-aqueous solution at a ratio of 1:10. The plant slurry underwent maceration in a shaking incubator (NB-205VL, N-BIOTEK, Korea) at 25°C with continuous shaking at 175 rpm for 24 h. The soluble liquid portion was separated from the marc using Whatman No. 1 filter paper (GE Health Care, UK) and a vacuum pump, resulting in the collection of CE micelles. The marc underwent two additional extractions following the same procedure, and all the micelles were combined before evaporating the ethanol using a vacuum rotary evaporator (RV 10 digital, IKA, Germany). The concentrated crude micelles were dried using a freeze dryer (Chris Alpha 2–4 LSCplus, Martin Christ Gefriertrocknungsanlagen GmbH, Germany). The resulting dried extract, obtained as a CE, was then assessed for its antiviral activity.

#### Liquid–liquid fractionation of crude extract

CEs demonstrating anti-SARS-CoV-2 activity with a log reduction of viral numbers ≥ 2.5 (≥ 99.7% efficacy) underwent further fractionation through a liquid–liquid separation technique employing water (a highly polar liquid) and ethyl acetate (a less polar liquid) [38]. The ratio of CE to both solvents was 1:10:10 (w/v/v). Initially, the CE was dissolved in water and transferred into a separating funnel. Subsequently, ethyl acetate was added, and the mixture was gently mixed and allowed to stand until two distinct phases of separation became evident. The upper phase (ethyl acetate-soluble phase) was separated from the lower phase (water-soluble phase) and collected in a new tube. The water-soluble phase underwent two additional rounds of fractionation by adding fresh ethyl acetate, and all the collected ethyl acetate-soluble portions were combined following a previously described process. EF and WF of individual CE were dried using a speed vacuum centrifuge (ScanSpeed, Labogene, Denmark) and freeze dryer, respectively. Both EFs and WFs were subsequently evaluated for their anti-SARS-CoV-2 activities.

### Antiviral assay

#### Cell culture

In the preliminary screening involving all 22 CEs using PEDV (clinically isolated) as a surrogate, Vero cells (CCL-81, ATCC, USA) were the host cells for PEDV propagation and the anti-PEDV assay. Subsequently, in the study of anti-SARS-CoV-2 activity, Vero E6 cells (CRL-1586, ATCC, USA) were employed as host cells for SARS-CoV-2 propagation, cytotoxicity assessments, and anti-SARS-CoV-2 assays.

Vero cells (CCL-81) were cultured in Dulbecco’s modified Eagle’s medium (DMEM; Gibco, USA), while Vero E6 cells (CRL-1586) were cultured in minimum essential medium (MEM; Gibco, USA). Both culture media were supplemented with 10% fetal bovine serum (FBS), 100 U/mL penicillin–streptomycin, and 1% GlutaMAX, all procured from Thermo Fisher Scientific (Life Technologies, USA). Cells were maintained at 37°C in a humidified incubator with 5% CO_2_.

#### Preparation of PEDV and SARS-CoV-2

PEDV was propagated in Vero cells (CCL-81) by culturing in serum-free DMEM supplemented with 10 μg/μL L-1-tosylamido-2-phenylethyl chloromethyl ketone (TPCK)-treated trypsin (Trypsin 1:250, Gibco) and 0.3% tryptose phosphate broth (Sigma-Aldrich), referred to as the infection medium. Once the cytopathic effect (CPE) reached 70%–80%, the viral stock was quantified for viral titer using a plaque assay and expressed as plaque-forming units per mL (pfu/mL) [39]. Further details of the methodology can be found in Additional File 1 of Supplementary Information (SI) 1.

For SARS-CoV-2, the Delta B.1.617.2 variant was isolated from nasopharyngeal swabs of a confirmed COVID-19 patient in Thailand and authenticated by the Tropical Medicine Diagnostic Reference Laboratory at the Faculty of Tropical Medicine, Mahidol University. The virus was then propagated in Vero E6 cells (CRL-1586) at a concentration of 2.5 × 10^5^ cells/mL in MEM supplemented with 2% fetal calf serum and 1% penicillin–streptomycin. To establish a high-titer viral stock, the virus was grown at 37°C in a 5% CO_2_ humidified incubator for 72 h. The viral titer was determined using TCID_50_, following the Reed–Muench method [40] and expressed as TCID_50_/mL. All experiments involving the live SARS-CoV-2 virus were conducted strictly at a certified BSL3 facility at the Faculty of Veterinary Science (Approval No. MU2023-038), Mahidol University.

#### Cytotoxicity assay

The cytotoxicity of the extracts/drugs on host cells was assessed using the 3-(4,5-dimethylthiazol-2-yl)-2,5-diphenyltetrazolium bromide (MTT) assay. The extracts/drugs were prediluted to stock concentrations in 0.5% dimethyl sulfoxide (DMSO, Merck) and further serially twofold diluted with the culture medium, covering a 1–1,000 μg/mL range.

Vero cells were seeded in 96-well plates (100 μL/well at a density of 2 × 10^5^ cells/mL) and incubated at 37°C for 24–48 h in a humidified incubator with 5% CO_2_ to achieve confluent monolayers. Furthermore, the cells were treated with various concentrations of herbal extracts/drugs in triplicates for 1 h (for the pre-entry study of the antiviral assay) and 48 h (for the postinfection treatment study of the antiviral assay). Extract-free culture medium served as a negative control. Cell viability was assessed using the MTT assay [20]. Briefly, the medium was replaced with the culture medium containing 0.5 mg/mL MTT (Sigma-Aldrich, USA) and incubated at 37°C for 2 h in a humidified incubator with 5% CO_2_. The MTT-medium solution was discarded, and formazan crystals were dissolved in 200 μL/well of DMSO before measuring the absorbance at 595 nm using a microplate reader (Sunrise™, Tecan Trading AG, Switzerland). Cell viability percentages were determined by normalization to the negative control, and the 50% cytotoxic concentration (CC_50_) was calculated using Microsoft Excel. Concentrations of extracts/drugs that resulted in more than 70% cell viability were considered maximum noncytotoxic concentrations (MNTCs) [41]. To evaluate their effectiveness against PEDV, CEs were tested at their MNTCs (single dose). The in vitro anti-SARS-CoV-2 activity of extracts/drugs was evaluated at three different concentrations, including their defined MNTCs and two consecutive twofold dilution concentrations [42].

#### Preliminary screening: antiviral activity of 22 CEs against PEDV as a surrogate

Twenty-two CEs underwent a preliminary screening to assess their antiviral potential using PEDV as a surrogate. A single-dose concentration equivalent to the defined MNTC of each CE was applied for the virucidal efficacy assay. In summary, PEDV was exposed to each CE for 5 min, followed by detecting any remaining infectious PEDV in Vero cells through the plaque reduction neutralization test (PRNT) [39]. The viral quantity was quantified and expressed in pfu/mL. The virucidal efficacy of the CE was calculated by comparing the reduction in plaque formation in the CE-treated PEDV to the untreated virus and reported as a percentage of viral reduction. Please refer to Additional File 1 (SI 2) for a comprehensive understanding of the methodology. CEs that demonstrated a minimum of 70% anti-PEDV efficacy were subjected to in vitro anti-SARS-CoV-2 activity studies.

#### In vitro anti-SARS-CoV-2 activity assay

Two potential modes of action for the anti-SARS-CoV-2 activity of herbal extracts were investigated in Vero E6 cells, following Abd-Alla et al. [43] and Kanjanasirirat et al. [16], with slight modifications. The first approach involved a pre-entry study where SARS-CoV-2 was directly exposed to the extracts/drugs, suggesting a direct effect on inactivating the virus viability (expressed as % virucidal). The second approach was a postinfection treatment study, in which host cells were first infected with SARS-CoV-2 before the application of extracts/drugs, indicating the ability to inhibit viral replication (expressed as % inhibition). The herbal extracts were tested at three concentrations, including their MNTCs, and two consecutive serial twofold dilution concentrations. All viral experiments were conducted in triplicate, and the results are presented as the mean ± standard deviation (SD).

Vero E6 cell monolayers were established by seeding 2 × 10^5^ cells/well (100 μL) in a 96-well plate and maintained at 37°C for 24 h in a 5% CO_2_ humidified incubator. Subsequently, the culture supernatant was removed, and Vero E6 cell monolayers were washed with phosphate-buffered saline (PBS) before being subjected to the corresponding anti-SARS-CoV-2 assay. Molnupiravir (EIDD-2801, Selleckchem, USA) was a positive control, while extract-free MEM was a negative control.

##### Pre-entry study

The extracts/drugs were assessed for their virucidal activity against SARS-CoV-2 following the American Society for Testing and Materials (ASTM) method no. ASTM E1053-20 [44] at three different concentrations based on their specific cytotoxicity results during a 1-h contact period. SARS-CoV-2 (1 × 10^5^ TCID_50_/mL) was exposed to the extracts/drugs solution at 37°C for 1 h. To observe virucidal activity, the tested virus suspension was cultured on a monolayer of Vero E6 cells with MEM (2% FBS) in a 96-well plate, and viral absorption was allowed for 2 h. The cells were washed twice with PBS before being replaced with a fresh medium. Culturing continued for 48 h before the observation of the CPE. The anti-SARS-CoV-2 efficacy of the extracts, as exhibited the reduction in viral number at the tested concentration, was calculated and expressed as % virucidal and an LRV compared to the negative control. LRVs of 2, 3, and 4 corresponded to viral reductions of 99%, 99.9%, and 99.99%, respectively. The calculations for percent viral reduction and LRV followed the method of Bullen et al. [45], with full details of the calculations provided in Additional File 1 (SI 3).

##### Postinfection treatment study

In a 96-well plate, a monolayer of Vero E6 cells was infected with SARS-CoV-2 at a concentration of 1 × 10^5^ TCID_50_/ml. The virus was allowed to absorb at 37°C for 2 h, after which the culture supernatant was removed. The infected cell monolayer was washed twice with sterile PBS and replaced with fresh MEM (2% FBS) containing various concentrations of herbal extracts/drugs. The culture was maintained at 37°C for 48 h in a humidified incubator with 5% CO_2_, and subsequently, the CPE was observed. The negative control exhibited the most significant CPE. The anti-SARS-CoV-2 activity in this approach was quantified as the % inhibition, which is also expressed as an LRV.

##### Half-maximal inhibitory concentration (IC_50_) evaluation

The IC_50_ values for molnupiravir and the most potent antiviral extract were determined using the TCID_50_ method, following the modified Reed–Muench method [40]. The most promising herbal extract and molnupiravir were assessed in both modes of action at their MNTCs and at four subsequent twofold serial dilution concentrations. Once the CPE was observed, the TCID_50_ was evaluated, and their respective IC_50_ concentrations were calculated.

### Evaluation of antiviral lead compounds from the most promising extract

The most promising extract was used to identify its antiviral lead compounds through LC–HRMS and molecular docking studies. However, the complexity of phytochemical separation on the herbal extract’s LC column always presents challenges due to insufficiently resolved separation, leading to the misannotation of these small molecules. To aid lead compound identification, the workflow for the evaluation of antiviral lead compounds was divided into four parts: *(1)* simplifying the complexity of WF-MLCE by partially separating it into several major fractions using CC, *(2)* identifying the tentative small molecules comprised in each CC fraction using LC–HRMS, *(3)* refining the numbers of the antiviral leads by determining anti-SARS-CoV-2 efficacy and total phenolic/flavonoid contents of the CC fractions, and *(4)* molecular docking of the compounds in the CC fractions that exhibited the highest anti-SARS-CoV-2 efficacy against SARS-CoV-2 M^pro^.

#### Fourier-transform infrared spectroscopic analysis (FTIR) of the most promising extract

Before the partial separation of the target extract by CC, FTIR spectroscopy was employed to characterize the target’s finger printing, so an appropriate column resin was consequently chosen. The FTIR spectra of the selected extracts were obtained in the mid-infrared (MIR) region (4000–400 cm^−1^) using a Bruker Tensor 27 FTIR spectrometer. An attenuated total reflectance accessory equipped with a diamond crystal was employed to collect spectra in reflection mode. Absorbance data were collected by accumulating 64 scans at a resolution of 4 cm^−1^. Baseline correction and spectral averaging were performed using Optical User Software (OPUS) 7.5 (Bruker Optics Ltd., Ettlingen, Germany). A reference spectrum of air was recorded as a baseline before conducting the sample measurement [46, 47].

#### Partial separation of the most promising extract using CC

Based on the overall chemical profiling of the selected extract using FTIR, Sephadex-LH20 resin (Cytiva, USA) was chosen for CC separation. The CC was conducted on an ÄKTA prime liquid chromatography system with a fraction collector (Amersham Biosciences, UK). The selected extract (250 mg/mL in water) was loaded onto the column. The sequential elution was conducted with a slight modification from previous studies [48–51] in the following steps: (1) 250 mL of water, (2) 250 mL of a 50% ethanolic-aqueous solution, and (3) 150 mL of 100% ethanol, at a flow rate of 2 mL/min. Eluent fractions (10 mL each) were collected and combined into the main fractions based on their absorbance peaks at 280 nm. Fractions were then freeze-dried prior to the phytochemical analysis using LC–HRMS. Additionally, the fractions’ polyphenolic contents, antioxidant activities, and anti-SARS-CoV-2 activities were determined (as detailed in Additional File 1 [SI 4]).

#### Identification of tentative small molecules of CC fractions (CCFs) using LC–HRMS

LC–HRMS was used to identify potential phytoconstituents in CCFs with adjusted conditions from previous studies [52–54]. An Ultimate 3000 RSLC system coupled with a Q Orbitrap high-resolution mass spectrometer (Thermo Fisher Scientific, Germany) was utilized for the analysis. LC separation was carried out on an XSelect HSS T3 column (2.1 × 100 mm, 2.5 µm, Waters, USA) at 30°C. Mobile phase A consisted of 0.1% formic acid (Optima LC–MS, Fisher Chemical) in an aqueous solution, while mobile phase B comprised 0.1% formic acid in acetonitrile (Optima LC–MS, Fisher Chemical). The gradient elution began at 1% B and increased to 95% B within 52 min at a 0.3 mL/min flow rate. The mass spectrometer was operated with heated electrospray ionization in positive and negative ion modes. A full-scan MS was conducted in the range of 100–1500 m/z with a resolution of 140,000. A data-dependent MS^2^ (dd-MS^2^) analysis was performed with a collision energy of 35 NCE with a resolution of 35,000. Data were acquired using Xcalibur software (version 4.3.73.11, Thermo Fisher Scientific, Germany). Data were processed using the Compound Discoverer 3.3 program (Copyright 2014–2022, Thermo Fisher Scientific, Germany). Tentative compounds were identified by comparing them with an mzCloud MS^n^ database (Thermo Fisher Scientific, Inc.), with a matching confidence of ≥ 50% for MS^2^ fragment patterns and ≥ 70% matching of precursor ions and their isotopic patterns, using the ChemSpider database (Royal Society of Chemistry) as the primary criteria. The compounds under consideration were identified along with their estimated abundances based on the peak area derived from the most prevalent adduct intensity.

#### Refining the identification of antiviral leads: Anti-SARS-CoV-2 assay and total phenolic/flavonoid contents

CCFs were tested for anti-SARS-CoV-2 activities following previously described protocols. Total phenolic and flavonoid contents and their antioxidant activities were evaluated (detailed in Additional File 1 [SI 4]). To shortlist the antiviral leads with the potent inhibitory activity, only the phytochemical constituents of the CCF(s) that exhibited outstanding anti-SARS-CoV-2 activities were subjected to molecular docking analysis.

#### In silico molecular docking: Prediction of potential inhibitors for the M^pro^ of SARS-CoV-2

Molecular docking analysis of small molecules in the target CCF(s) against the structure of the SARS-CoV-2 M^pro^ was performed using MGLTools (AutoDockTools [ADT] 1.5.6 module) [55]. We also examined three repurposed drugs as references: lopinavir, hydroxychloroquine, and molnupiravir. The crystal structure of SARS-CoV-2 M^pro^ was obtained from Protein Data Bank (PDB ID: 6LU7) [56] and processed in PDB format. The phytocompound structures, serving as ligands, were retrieved from NCBI PubChem database and MolView [57]. Ligand structures were extracted as 3D conformers in SDF or MOL format and then converted to PDB format using Open Babel GUI [58] before conducting molecular docking with ADT module software [59]. The grid box for the active site of M^pro^ was defined with dimensions of X = 66 Å, Y = 70 Å, and Z = 48 Å, and the center was positioned at X =  − 10.357 Å, Y = 18.601 Å, and Z = 67.669 Å. This grid box was centered around residues His-41, Asn-142, Gly-143, Ser144, Cys-145, His-163, Met-165, and Glu-166 [60, 61]. Ligand preparation was carried out using the Torsion tree function with rotatable bonds. The docking conformations of ligand-SARS-CoV-2 M^pro^ were predicted at pH 7, and binding energies were expressed in kcal/mol, where a higher negative value of binding energy indicates a stronger binding affinity. Furthermore, we compared their inhibition constants (Ki), expressed in µM, among the ligand compounds. Ki represents the concentration at which a compound successfully inhibits 50% of viral protein functioning in silico. A lower Ki value indicates higher inhibitory activity against viral protein function. Compounds that exhibited distinctive docking scores were further assessed for their binding with the active site residues of SARS-CoV-2 in a 3D model using PyMOL [62].

#### Stability of the docked complex using MD simulation

The top three docked phytocompounds were investigated for their structural stability of the ligand–protein complexes by conducting an all-atom MD simulation for each docked complex. The all-atom MD simulation was performed utilizing a time step of 2 fs, employing the AMBER20 software package [63]. Parameters governing bonded and nonbonded interactions of all inhibitors were managed using the General Amber Force Field (GAFF) [64]. Protein parameters were defined using the AMBER ff14SB force field [65]. TIP3P water molecules [66] were used for system solvation, and Na^+^ counterions were included to maintain neutrality. The isobaric-isothermal (NPT) ensemble was employed, with a constant pressure of 1 atm and a temperature of 310 K. The SHAKE algorithm [67] was utilized to constrain all bonds involving hydrogen. The nonbonded interactions were computed with a residue-based cutoff of 12 Å. Long-range electrostatic interactions were handled using the particle mesh Ewald method [68]. The steepest descent method was used for 1000 iterations to reduce structurally unfavorable interactions, followed by 2000 iterations of conjugate gradient energy minimization on the complex structure. Furthermore, the system was gradually heated to 310 K over 100 ps. Restrained MD simulations were performed for a total of 5.0 ns, with decreasing restraints applied at intervals of 50, 30, 20, 10, 5, and 1 kcal/mol·Å^2^. Subsequently, unrestrained MD simulations were conducted for 500 ps, followed by MD simulations in the NPT ensemble (1 atm and 310 K) without restraints until reaching 50 ns. The CPPTRAJ module of AMBER20 was employed to calculate structural and dynamic properties, including the root-mean-square deviation (RMSD) of the protein–ligand complex, the radius of gyration (Rg) of the protein–ligand complex, and the number of atom contacts (#Contacts) within 3.5 Å of the ligand.

### Statistical analysis

All experiments were repeated in triplicate, and the results are given as mean ± SD using Microsoft Excel. Statistical analysis was conducted using SPSS (IBM SPSS Statistics version 26, IBM Corporation 2019).

## Results

### Screening for promising anti-SARS-CoV-2 candidates using in vitro antiviral assay

#### Preliminary screening of potential antiviral CEs using PEDV as a surrogate

To explore a new promising Thai medicinal plant candidate as an anti-SARS-CoV-2 agent, we rapidly scrutinized 22 ethanolic-aqueous CEs from 19 plants for their potential antiviral activity against PEDV to overcome the containment capabilities of BSL3. The results of anti-PEDV efficacies tested with a single dose (MNTC) of individual CE identified eight potential CEs that demonstrated potential antivirus performance, as defined by achieving ≥ 70% virucidal efficacy (Table S2 in Additional File 2). Eight CEs met this criterion: S1: *Colubrina asiatica* (L.) Brongn.; S2: *Morus alba* Linn. (leaves) (89%); S3: *Gynostemma pentaphyllum* (Thunb) Makino; S4: *Artemisia annua* L.; S5: *Centella asiatica* (L.); S6: *Justicia gendarussa* Burm. f.; S7: *Helicteres isora* L.; and S8: *Phyllanthus niruri* L. Consequently, these eight CEs were selected for further investigation on their antiviral performances with SARS-CoV-2 using Vero E6 cells as the host cells.

#### Anti-SARS-CoV-2 activity of potential herbal CEs

The cytotoxicity of nine CEs in Vero E6 cells and their CC_50_ are presented in Fig. S2 (Additional File 2). The results of the 1-h contact period assay (conducted for the pre-entry study) generally indicated a similar trend of moderate cytotoxicity across all extracts, with CC_50_ values ranging from 0.24 to 0.40 mg/mL. Notable exceptions were observed in S3 and S8-D, which exhibited the lowest cytotoxicity, with a CC_50_ exceeding 1 mg/mL (Fig. S2a). A similar cytotoxicity trend was observed in the 48-h contact period (conducted for the postinfection treatment study), with CC_50_ values spanning a slightly wider range (0.30–0.71 mg/mL) (Fig. S2b). Most CEs demonstrated anti-SARS-CoV-2 efficacy with an LRV of 2 (~ 99% virucidal efficacy) in the pre-entry mode (Table S3). The highest efficacy was observed in S8-D with an LRV of 2.56 (99.68% virucidal) at a 0.0625 mg/mL concentration. Meanwhile, significant anti-SARS-CoV-2 activity with an LRV of ~ 2.5 (99.7% viral inhibition) was observed in four CEs (S1, S2, S8-L, and S8-D) in the postinfection treatment mode. The most potent inhibition activity was also recorded for S8-D at 0.1250 mg/mL (2.94 LRV and 99.89% inhibition), followed by S2 at 0.0313 mg/mL (2.60 LRV and 99.75% inhibition).

**Fig. 1:**
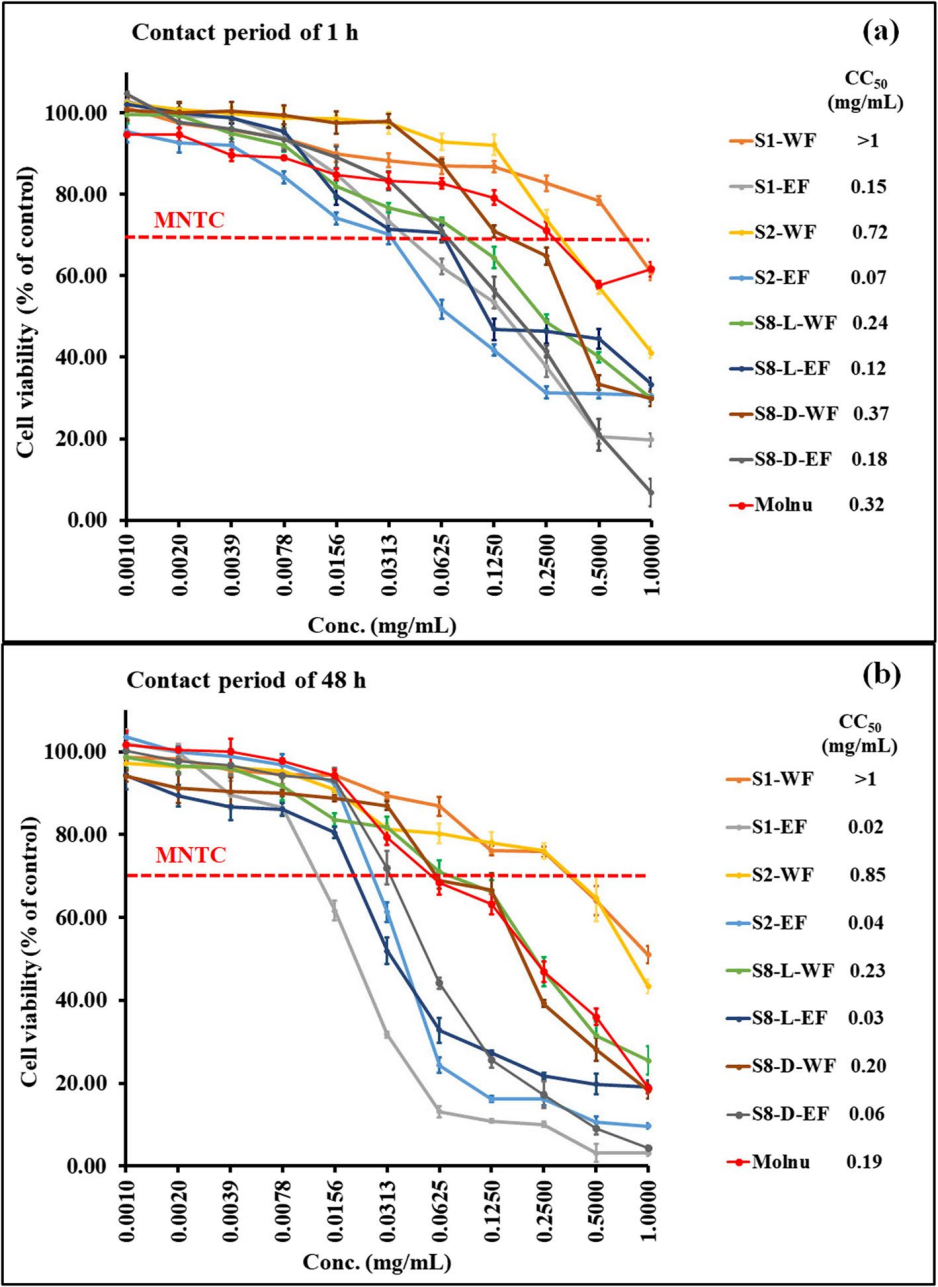
Cytotoxicity of water fractions (WF) and ethyl acetate fractions (EF) of the selected four crude extracts and molnupiravir (Molnu) evaluated over Vero E6 cells using the 3-(4,5-dimethylthiazol-2-yl)-2,5-diphenyltetrazolium bromide (MTT) assay. Results are expressed as % cell viability (mean ± SD) at various extract concentrations with CC_50_. **a** After 1 h of treatment with fractions/drugs, and (**b**) after 48 h of treatment with fractions/drugs. MNTC line indicates the maximum nontoxic concentration with 70% cell viability of individual fraction

The four most promising CEs (S1, S2, S8-L, and S8-D) underwent liquid–liquid fractionation using a water and ethyl acetate solvent system. The cytotoxicity analysis of the resulting WFs and EFs showed that the WFs had significantly lower cytotoxicity to Vero E6 cells than EFs (Fig. 1). Among all fractions, S1-WF and S2-WF demonstrated very low cytotoxicity, with a CC_50_ of ≥ 0.72 mg/mL for both 1-h and 48-h contact periods. Their CC_50_ also showed 2–4 times lower cytotoxicity than molnupiravir.

For anti-SARS-CoV-2 performance, molnupiravir achieved 99.81% virucidal activity (LRV of 2.76) in the pre-entry mode and 99.08% inhibition of viral replication (LRV of 2.05) in the postinfection treatment mode at a concentration of 0.0625 mg/mL for both phases (Table 1).

**Table 1:** Anti-SARS-CoV-2 efficacy of molnupiravir

Pre-entry study			Postinfection treatment study		
[Molnupiravir] (mg/mL)	Anti-SARS-CoV-2 efficacy		[Molnupiravir] (mg/mL)	Anti-SARS-CoV-2 efficacy	
	Log Reduction	% Virucidal		Log Reduction	% Inhibition
0.0625	2.76 ± 0.21	99.81 ± 0.09	0.0625	2.05 ± 0.08	99.08 ± 0.16
0.0313	2.55 ± 0.20	99.69 ± 0.13	0.0313	1.93 ± 0.09	98.77 ± 0.27
0.0156	2.22 ± 0.26	99.30 ± 0.36	0.0156	1.84 ± 0.12	98.48 ± 0.48
0.0078	2.05 ± 0.23	99.02 ± 0.41	0.0078	1.55 ± 0.20	96.89 ± 1.30
0.0039	1.76 ± 0.18	98.11 ± 0.85	0.0039	1.47 ± 0.06	96.47 ± 0.74

**Fig. 2:**
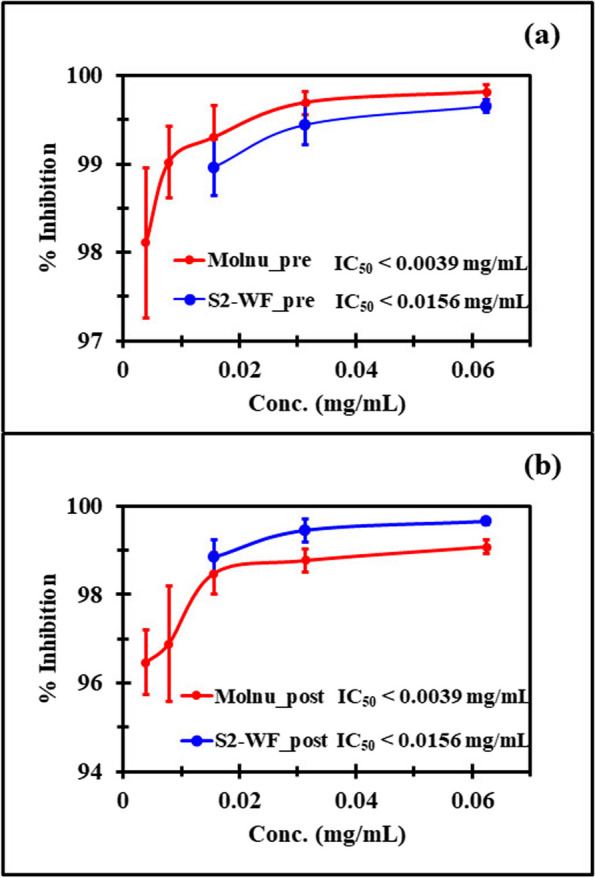
Anti-SARS-CoV-2 activities and the IC_50_ of molnupiravir (Molnu) and S2-WF evaluated over Vero E6 cells. **a** Comparison of their antiviral activities and IC_50_ in pre-entry mode, and (**b**) comparison of their antiviral activities and IC_50_ in postinfection treatment mode

The anti-SARS-CoV-2 activity of WFs and EFs was observed in the same fashion as molnupiravir by exhibiting greater antiviral efficacy in the pre-entry phase than that in the postinfection phase, which was in contrast with the CE patterns (Table 2). The fractions also showed slightly higher virucidal efficacy than the original CE. Among all the fractions, S2-WF demonstrated exceptional virucidal efficacy, achieving an LRV of 3.06 (99.9% virucidal) at a 0.250 mg/mL concentration. S2-WF also exhibited the highest efficacy in the postinfection phase, with an LRV of 2.90 (99.8% inhibition of viral replication) at concentrations of 0.1250 and 0.250 mg/mL. The IC_50_ of S2-WF was lower than 0.0156 mg/mL for both approaches, while the IC_50_ of molnupiravir was lower than 0.0039 mg/mL (Fig. 2). Due to certain limitations in determining the IC_50_ of S2-WF in the anti-SARS-CoV-2 test, the lowest concentration was only diluted to 0.0156 mg/mL. Nevertheless, based on the overall trend, the IC_50_ of S2-WF appears to be as low as that of molnupiravir. Therefore, S2-WF, as WF-MLCE, was appointed as the most promising anti-SARS-CoV-2 agent in this research, which was then evaluated for its antiviral lead compounds and in silico potential interactions of those compounds against the M^pro^ of SARS-CoV-2.

**Table 2:** Anti-SARS-CoV-2 efficacy of water fraction (WF) and ethyl acetate fraction (EF) obtained from liquid–liquid fractionation of the four crude extracts (CEs)

Extract	Pre-entry study						Postinfection treatment study					
	Conc (mg/mL)		Anti-SARS-CoV-2 efficacy				Conc. (mg/mL)		Anti-SARS-CoV-2 efficacy			
			Log Reduction		% Virucidal				Log Reduction		% Inhibition	
S1-WF	0.5000		2.52 ± 0.14		99.71 ± 0.05		0.500		1.64 ± 0.27		97.63 ± 1.03	
	0.250		2.35 ± 0.30		99.53 ± 0.24		0.2500		1.73 ± 0.20		98.05 ± 0.94	
	0.1250		2.27 ± 0.30		99.44 ± 0.23		0.1250		1.98 ± 0.69		98.28 ± 1.29	
S1-EF	0.0313		2.69 ± 0.22		99.79 ± 0.08		0.0313		2.31 ± 0.38		99.45 ± 0.27	
	0.0156		2.44 ± 0.23		99.62 ± 0.20		0.0156		2.40 ± 0.33		99.54 ± 0.27	
	0.0078		2.35 ± 0.14		99.56 ± 0.17		0.0078		2.44 ± 0.07		99.65 ± 0.07	
S2-WF	0.250		3.06 ± 0.14		99.92 ± 0.02		0.2500		2.90 ± 0.07		99.88 ± 0.03	
	0.1250		2.65 ± 0.25		99.77 ± 0.10		0.1250		2.90 ± 0.07		99.88 ± 0.03	
	0.0625		2.44 ± 0.07		99.65 ± 0.07		0.0625		2.48 ± 0.30		99.63 ± 0.23	
S2-EF	0.0313		2.44 ± 0.07		99.65 ± 0.07		0.0313		2.02 ± 0.12		99.04 ± 0.37	
	0.0156		2.35 ± 0.47		99.04 ± 0.59		0.0156		2.10 ± 0.18		99.17 ± 0.34	
	0.0078		2.10 ± 0.22		99.21 ± 0.26		0.0078		2.35 ± 0.35		99.46 ± 0.38	
S8-L-WF	0.0625		2.44 ± 0.07		99.65 ±0.07		0.0625		2.19 ± 0.14		99.37 ± 0.11	
	0.0313		2.35 ± 0.30		99.53 ±0.24		0.0313		1.85 ± 0.12		98.65 ± 0.23	
	0.0156		2.10 ± 0.22		99.21 ±0.26		0.0156		1.81 ± 0.25		98.34 ± 1.03	
S8-L-EF	0.0625		2.44 ± 0.48		99.51 ± 0.42		0.0625		2.02 ± 0.12		99.04 ± 0.37	
	0.0313		2.31 ± 0.38		99.45 ± 0.27		0.0313		1.94 ± 0.21		98.85 ± 0.38	
	0.0156		2.44 ± 0.07		99.53 ± 0.24		0.0156		1.85 ± 0.14		98.65 ± 0.23	
S8-D-WF	0.1250		1.94 ± 0.10		98.85 ± 0.38		0.1250		1.56 ± 0.22		97.26 ± 0.85	
	0.0625		2.69 ± 0.22		99.79 ± 0.08		0.0625		1.52 ± 0.22		96.89 ± 1.30	
	0.0313		2.65 ± 0.25		99.77 ± 0.10		0.0313		1.44 ± 0.07		96.47 ± 0.74	
S8-D-EF	0.0625		2.85 ± 0.14		99.86 ± 0.02		0.0625		2.31 ± 0.38		99.45 ± 0.27	
	0.0313		2.52 ± 0.14		99.71 ± 0.05		0.0313		2.27 ± 0.30		99.44 ± 0.23	
	0.0156		2.35 ± 0.30		99.53 ± 0.24		0.0156		2.19 ± 0.14		99.37 ± 0.11	

Results are expressed as mean ± SD of quadruplicate experiments. *WF* water extract, *EF* ethyl acetate extract; Conc., tested concentration of extract; Log reduction, the reduction of viral titer after experimental treatment in logarithmic function; % Inhibition or % Virucidal, % viral titer reduction by comparing with the initial viral load. S1, S2 and S8, herbal names were listed as described in Table S1

### Evaluation of antiviral lead compounds in the most promising extract: Water fraction of mulberry leaf crude extract (WF-MLCE)

#### Finger printing of WF-MLCE by FTIR spectroscopy

**Fig. 3:**
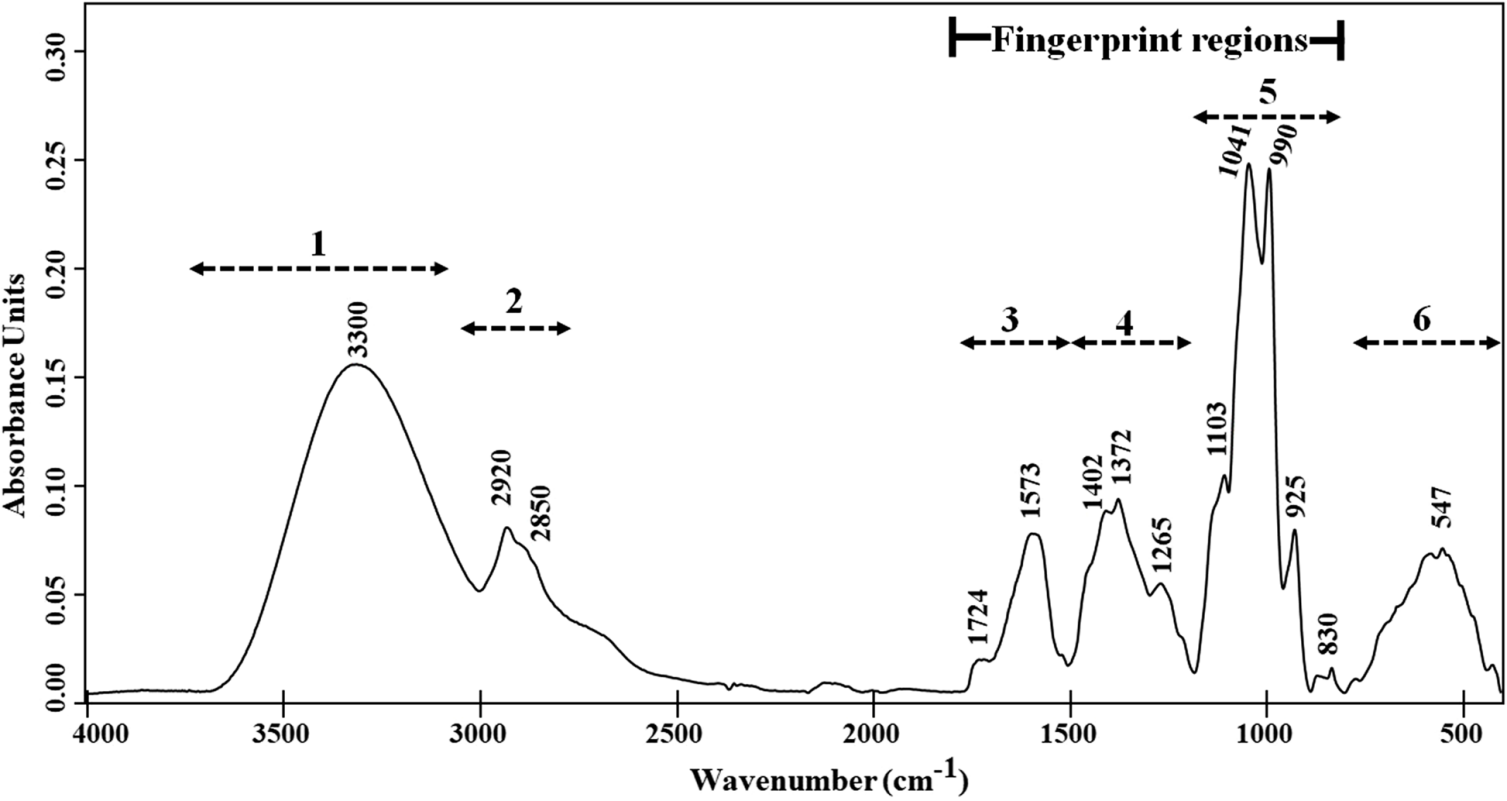
FTIR spectrum of water fraction of mulberry leaf crude extract (S2-WF). The spectral assignment of each region can be found in Table S4 (Additional File 2)

Figure 3 illustrates the FTIR spectral profile as finger printing of WF-MLCE, with six distinct regions of the spectrum defined to characterize its containing compound classes. The spectral band assignments are also detailed in Table S4 (Additional File 2). Region 1, 2 and 6 encompass general functional groups that may not necessarily serve as characteristic bands for classifying compounds in herbal extracts. However, the presence of these spectral bands is likely integral to the structural composition of polyphenolics, carbohydrates, and lipids [47, 69–72].

Regions 3–5 are commonly recognized as fingerprint regions for polyphenolics [70, 72]. The peaks at 1573 cm^−1^, alongside those in the 1500–1450 cm^−1^ range, could be attributed to the C = C–C skeleton and C–H bonds of aromatic rings, including phenols [71]. The noticeable coabsorbances detected at 1540–1140 cm^−1^ were typically associated with the characteristics of flavonoids, including C = O, and C = C bonds of the phenol group, as well as –C–OH, C–H, and O–H deformation of the aromatic ring [47, 69, 72]. In addition, distinct bands appeared at 1041 cm^−1^ and 990 cm^−1^, accompanied by a smaller band at 1103 cm^−1^, suggesting the sugar content within the molecules. The bands attributed to the C–H of Ring B in the flavonoid structure (1100–1075 cm^−1^ range) were also observed [69, 71, 72]. Our FTIR spectral profile of WF-MLCE strongly suggested a dominant presence of flavonoids, particularly flavonols and flavonoid glycosides, along with a moderate presence of phenolic and carboxylic acid compounds.

#### Partial separation of WF-MLCE by CC

In total, five chromatographic fractions (F1–F5) were obtained from the CC of WF-MLCE. F1–F4 were obtained through elution with water, while F5 was obtained with a 50% ethanolic-aqueous solution (Fig. S3 in Additional File 2). Notably, no absorbance peak response was observed when eluting with absolute ethanol. F1–F5 were then underwent the identification of antiviral lead compounds, including anti-SASR-CoV-2 efficacy testing, antioxidant assay, LC–HRMS, and molecular docking analysis.

#### Tentative phytochemical identification of CC fractions of WF-MLCE by LC–HRMS

The five fractions were subjected to LC–HRMS to tentatively identify their chemical constituents. Various classes of compounds were found, including polyphenolics, sugars and their derivatives, amino/organic acids, and peptides. A total of 214 putative compounds were identified. Table 3 presents the list of tentative compounds found in F1 (19 compounds). The two most prevalent polyphenolics found in this fraction, 4-O-caffeoylquinic acid and trans-5-O-caffeoylquinic acid. Additionally, benzoyl derivative compounds and amino acid and amino-sugar groups were also observed.

**Table 3:** LC–HRMS analysis of tentative phytochemical contents of F1 of WF-MLCE (S2-WF)

No	Tentative phytocompound	Formula	Theoretical MW	Reference ion	Average Peak Area (from Most Common Adduct)
1	Adenine	C_5_H_5_N_5_	135.0545	[M + H]^+^	1.17E + 08
2	UDP-N-acetylglucosamine	C_17_H_27_N_3_O_17_P_2_	607.0816	[M-H]^−^	1.03E + 08
3	5-O-Caffeoylquinic acid	C_16_H_18_O_9_	354.0951	[M-H]^−^	8.53E + 07
4	4-O-Caffeoylquinic acid	C_16_H_18_O_9_	354.0951	[M-H]^−^	3.36E + 07
5	L-Pyroglutamic acid	C_5_H_7_NO_3_	129.0426	[M + H]^+^	4.90E + 07
6	Proline	C_5_H_9_NO_2_	115.0633	[M + H]^+^	3.70E + 07
7	Acetylcholine	C_7_H_15_NO_2_	145.1103	[M + H]^+^	3.61E + 07
8	Υ-L-Glutamyl-L-glutamic acid	C_10_H_16_N_2_O_7_	276.0958	[M + H]^+^	2.59E + 07
9	1-Stearoylglycerol	C_21_H_42_O_4_	358.3083	[M + H]^+^	2.37E + 07
10	2,6-Di-tert-butyl-1,4-benzoquinone	C_14_H_20_O_2_	220.1463	[M + H]^+^	1.65E + 07
11	9-Oxo-10(E),12(E)-octadecadienoic acid	C_18_H_30_O_3_	294.2195	[M + H]^+^	1.47E + 07
12	12-oxo Phytodienoic Acid	C_18_H_28_O_3_	292.2038	[M + H]^+^	1.35E + 07
13	4-Hydroxybenzaldehyde	C_7_H_6_O_2_	122.0368	[M + H]^+^	1.12E + 07
14	Estriol	C18H24O3	288.1725	[M + H] +	8.49E + 06
15	L-Glutathione (reduced)	C10H17N3O6S	307.0838	[M + H] +	6.47E + 06
16	(2R,5R,6R)-3-[(1E,3E)-hepta-1,3-dien-1-yl]-5,6-dihydroxy-2-(hydroxymethyl)cyclohexan-1-one	C14H22O4	254.1518	[M + H] +	5.43E + 06
17	Jasmonic acid	C12H18O3	210.1256	[M-H]-	7.20E + 06
18	Gentisic acid 5-O-β-D-glucoside	C13H16O9	316.0794	[M-H]-	4.80E + 06
19	NP-014604	C19H34O10	422.2152	[M-H]-	3.17E + 06

Table 4 presents the list of tentative compounds identified in F5 (29 compounds). The most prominent compound in this fraction was 1-deoxynojirimycin (DNJ). Furthermore, F5 predominantly contained flavonoids and flavonoid glycosides, such as quercetin, kaempferol, myricetin, rutin, isoquercetin, quercetin 3,7-di-*O*-glucoside, astragalin, and kaempferol 3-*O*-rutinoside-7-*O*-rhamnoside. The tentative compound lists for F2–F4 are presented in Tables S5–S7 (Additional File 2), respectively. The predominant compound classes identified in these three fractions were amino acids, phenolic acids, carboxylic acids, and their derivatives. The chemical profiles tentatively obtained from the five fractions through LC–HRMS align with the FTIR spectral profile of WF-MLCE.

**Table 4:** LC–HRMS analysis of tentative phytochemical contents of F5 of WF-MLCE (S2-WF)

No	Tentative phytocompound	Formula	Theoretical MW	Reference ion	Average Peak Area (from Most Common Adduct)
1	1-deoxynorijimycin	C_6_H_13_NO_4_	163.0845	[M + H]^+^	5.95E + 09
2	Quercetin 3-O-rutinoside [Rutin]	C_27_H_30_O_16_	610.1534	[M-H]^−^	5.34E + 09
3	Kaempferol 3-O-neohesperidoside	C_27_H_30_O_15_	594.1585	[M-H]-	4.25E + 09
4	Quercetin 3,7-di-O-glucoside	C_27_H_30_O_17_	626.1483	[M-H]-	3.73E + 09
5	Quercetin-3β-D-glucoside [Isoquercetrin]	C_21_ H_20_O_12_	464.0955	[M-H]-	3.58E + 09
6	Quercetin 3-O-rutinoside-7-O-rhamnoside [Morkotin B]	C_33_H_40_O_20_	756.21129	[M-H]-	3.33E + 09
7	Kaempferol 3-O-rutinoside	C_27_H_30_O_15_	594.1585	[M-H]-	3.12E + 09
8	Quercetin 3-O-robinobioside [Bioquercetrin]	C_27_H_30_O_16_	610.1534	[M-H]-	2.75E + 09
9	Kaempferol 3,7-di-O-glucoside	C_27_H_30_O_16_	610.1534	[M-H]-	2.70E + 09
10.1^a^	Kaempferol 3-O-galactoside [Trifolin]	C_21_H_20_O_11_	448.1006	[M-H]-	2.23E + 09
10.2^a^	Kaempferol 3-O-glucoside [Astragalin]	C_21_H_20_O_11_	448.1006		
11	Kaempferol 3-O-rhamninoside	C_33_H_40_O_19_	740.2164	[M-H]-	2.18E + 09
12	Kaempferol 3-O-β-D-glucosylgalactoside	C27H30O16	610.1534	[M + H] +	1.47E + 09
13.1^b^	Robinin	C33H40O19	740.2164	[M + H] +	1.43E + 09
13.2^b^	Kaempferol 3-O-rutinoside-7-O-rhamnoside [Marakrol B]	C33H40O19	740.2164		
14	Quercetin	C15H10O7	302.0422	[M + H] +	1.41E + 09
15	Kaempferol	C15H10O6	286.0477	[M + H] +	1.28E + 09
16	Methyl cinnamate	C10H10O2	162.0681	[M + H] +	6.51E + 07
17	Luteolin-4'-glucoside	C21H20O11	448.1006	[M + H] +	5.18E + 07
18	Luteolin-7-glucoside [Cynaroside]	C21H20O11	448.1006	[M-H]-	2.05E + 07
19	Myricetin 3-O- β -D-galactopyranoside	C21H20O13	480.0904	[M-H]-	2.03E + 07
20	Thiamine	C12H16N4OS	264.1045	[M + H] +	1.53E + 07
21	Myricetin	C15H10O8	318.0371	[M + H] +	1.36E + 07
22	1-[4-hydroxy-3-(3-methylbut-2-en-1-yl) phenyl]ethan-1-one	C13H16O2	204.1150	[M + H] +	1.15E + 07
23	1,4-dihydroxyheptadec-16-en-2-yl acetate	C19H36O4	328.26076	[M + H] +	1.06E + 07
24	Formonetin	C16H12O4	268.0736	[M + H] +	8.06E + 06
25	4-((3S)-7-hydroxy-8-(3-methylbut-2-en-1-yl)-3,4-dihydro-2H-1-benzopyran-3-yl)benzene-1,3-diol	C20H22O4	326.1518	[M + H] +	7.00E + 06
26	Resveratrol	C14H12O3	228.0786	[M-H]-	5.20E + 06
27	Apigetrin	C21H20O10	432.1057	[M-H]-	4.65E + 06
28	Bayin	C21H20O9	416.1107	[M-H]-	4.24E + 06
29	2-(2-Oxo-8,9-dihydro-2H-furo(2,3-h) chromen-8-yl)-2-propanyl beta-D-glucopyranoside	C20H24O9	408.1420	[M-H]-	3.20E + 06

^a^The tentative compound could possibly identify either as one of them because both compounds are being isomer and their identification scores were similar

^b^The tentative compound could possibly identify either as one of them because both compounds are being isomer and their identification scores were similar

#### Refining the numbers of the antiviral lead candidates of CC fractions of WF-MLCE

The results of the antiviral activities of the five CC fractions can be found in Table S8, where their cytotoxicity can also be found in Fig. S4 (Additional File 2). F1 and F5 exhibited notably potent virucidal activity, achieving LRVs of > 3 (99.9% virucidal). F5 was measured with the highest total polyphenolic content and antioxidant activities. However, there was no significant distinction in viral inhibition efficiencies among the five fractions, with LRVs ranging between 2.0 and 2.8 in the postinfection treatment study. In our study scenario, there is no distinctive link between the total phenolic content, total flavonoid content, and antioxidant activities of F1–F5 (Table S9) and their anti-SARS-CoV-2 efficacy. Therefore, the promising virucidal efficacies of the CC fraction(s) were solely used as a criterion to target the antiviral lead fraction for further molecular docking analysis. Given that, the tentative compounds found in F1 and F5 were subjected to molecular docking.

#### In silico molecular docking

The binding affinities against the COVID-19 virus-M^pro^ of tentative compounds of F1 and F5, as well as of the repurposed drugs, are reported in Table 5 in terms of binding energy (kcal/mol) and the inhibition constant value (Ki; μM). Hydroxychloroquine and molnupiravir exhibited similar binding affinities (binding energies of − 6.09 and − 6.21 kcal/mol, respectively). Lopinavir displayed the lowest potency, with a binding energy of − 5.65 kcal/mol. The inhibition constants mirrored their binding energy results. The docking results for the 48 tentative phytocompounds revealed different binding affinities. The notable compounds in F1 were 5-*O*-caffeoylquinic acid (No. 3), 4-*O*-caffeoylquinic acid (No. 4), and estriol (No. 14), which exhibited binding energies of − 6.93 kcal/mol (Ki 8.30 μM), − 6.79 kcal/mol (Ki 10.59 μM), and − 6.96 kcal/mol (Ki 7.95 μM), respectively. However, their docking scores were not significantly better than those of the reference drugs.

**Table 5:** Molecular docking scores of drugs and tentative phytochemical contents of F1 and F5 with M^pro^ (6LU7) of SARS-CoV2

No.	Drug/Phytochemical	Docking Score	
		Binding energy	Inhibition constant [Ki]
		(kcal/mol)	(mM)
**Drugs **			
1	Lopinavir	-5.65	72.57
2	Hydroxychloroquine	-6.09	34.14
3	Molnupiravir	-6.21	28.19
** Fraction 1 **			
1	Adenine	-3.85	1510.00
2	UDP-N-acetylglucosamine	-4.84	285.00
3	trans-5-*O*-Caffeoylquinic acid	-6.93	8.30
4	4-*O*-Caffeoylquinic acid	-6.79	10.59
5	L-Pyroglutamic acid	-4.37	624.24
6	Proline	-4.89	261.92
7	Acetylcholine	-3.65	2090.00
8	Υ-L-Glutamyl-L-glutamic acid	-4.54	470.12
9	1-Stearoylglycerol	-5.58	81.76
10	2,6-Di-tert-butyl-1,4-benzoquinone	-5.37	116.65
11	9-Oxo-10(E),12(E)-octadecadienoic acid	-4.52	484.03
12	12-oxo Phytodienoic Acid	-5.14	171.92
13	4-Hydroxybenzaldehyde	-4.03	1120.00
14	Estriol	-6.96	7.95
15	L-Glutathione (reduced)	-5.28	135.89
16	(2R,5R,6R)-3-((1E,3E)-hepta-1,3-dien-1-yl)-5,6-dihydroxy-2-(hydroxymethyl) cyclohexan-1-one	-6.74	11.52
17	Jasmonic acid	-5.13	173.67
18	Gentisic acid 5-*O*-β-D-glucoside	-5.84	52.46
**Fraction 5**			
1	1-deoxynorijimycin	-4.57	444.76
2	Quercetin 3-*O*-rutinoside [Rutin]	-7.89	1.65
3	Kaempferol 3-*O*-neohesperidoside	-6.36	21.82
4	Quercetin 3,7-di-*O*-glucoside	-7.50	3.19
5	Quercetin-3β-D-glucoside [Isoquercetrin]	-7.62	2.59
6	Quercetin 3-*O*-rutinoside-7-*O*-rhamnoside [Morkotin B]	-6.10	33.81
7	Kaempferol 3-*O*-rutinoside [Nicotiflorin]	-7.04	6.91
8	Quercetin 3-*O*-robinobioside [Bioquercetrin]	-8.68	0.43
9	Kaempferol 3,7-di-*O*-glucoside	-7.86	1.73
10	Kaempferol 3-*O*-galactoside [Trifolin]	-8.10	1.15
11	Kaempferol 3-*O*-glucoside [Astragalin]	-7.41	3.68
12	Kaempferol 3-*O*-rhamninoside	-5.24	143.24
13	Kaempferol 3-*O*-β-D-glucosylgalactoside	-6.04	37.27
14	Kaempferol 3-*O*-robioside-7-*O*-rhamnoside [Robinin]	-5.43	105.35
15	Kaempferol 3-*O*-rutinoside-7-*O*-rhamnoside [Maragrol B]	-9.01	0.25
16	Quercetin	-6.91	8.61
17	Kaempferol	-7.38	3.89
18	Methyl cinnamate	-4.64	394.66
19	Luteolin-4'-glucoside	-7.54	2.99
20	Luteolin-7-glucoside [Cynaroside]	-7.51	3.11
21	Myricetin 3-*O*- β -D-galactopyranoside	-9.66	0.08
22	Thiamine	-5.82	54.60
23	Myricetin	-6.93	8.35
24	1-(4-hydroxy-3-(3-methylbut-2-en-1-yl) phenyl) ethan-1-one	-6.23	27.35
25	1,4-dihydroxyheptadec-16-en-2-yl acetate	-5.74	62.50
26	Formonetin	-6.96	7.97
27	4-((3S)-7-hydroxy-8-(3-methylbut-2-en-1-yl)-3,4-dihydro-2H-1-benzopyran-3-yl) benzene-1,3-diol	-6.91	8.56
28	Resveratrol	-6.51	16.99
29	Apigetrin	-7.82	1.84
30	Bayin	-8.65	0.46
31	2-(2-Oxo-8,9-dihydro-2H-furo(2,3-h) chromen-8-yl)-2-propanyl beta-D-glucopyranoside	-8.38	0.72

In contrast, F5 contained more promising compounds, particularly glycoside derivatives of flavonoids. Some of them displayed remarkable binding affinity with M^pro^. The top three most promising compounds were myricetin 3-*O*-β-D-galactopyranoside (No. 21, binding energy of − 9.66 kcal/mol and Ki of 0.08 μM), maragrol B (No. 15, binding energy of − 9.01 kcal/mol and Ki of 0.25 μM), and quercetin 3-*O*-robinobioside (No. 8, binding energy of − 8.68 kcal/mol and Ki of 0.43 μM). Rutin (No. 2), one of the highly abundant compounds in F5, also displayed a notable molecular docking score (binding energy of − 7.89 kcal/mol and Ki of 1.65 μM). Several other flavonoids, such as isoquercetin, trifolin, and kaempferol, also exhibited optimistic scores.

**Fig. 4:**
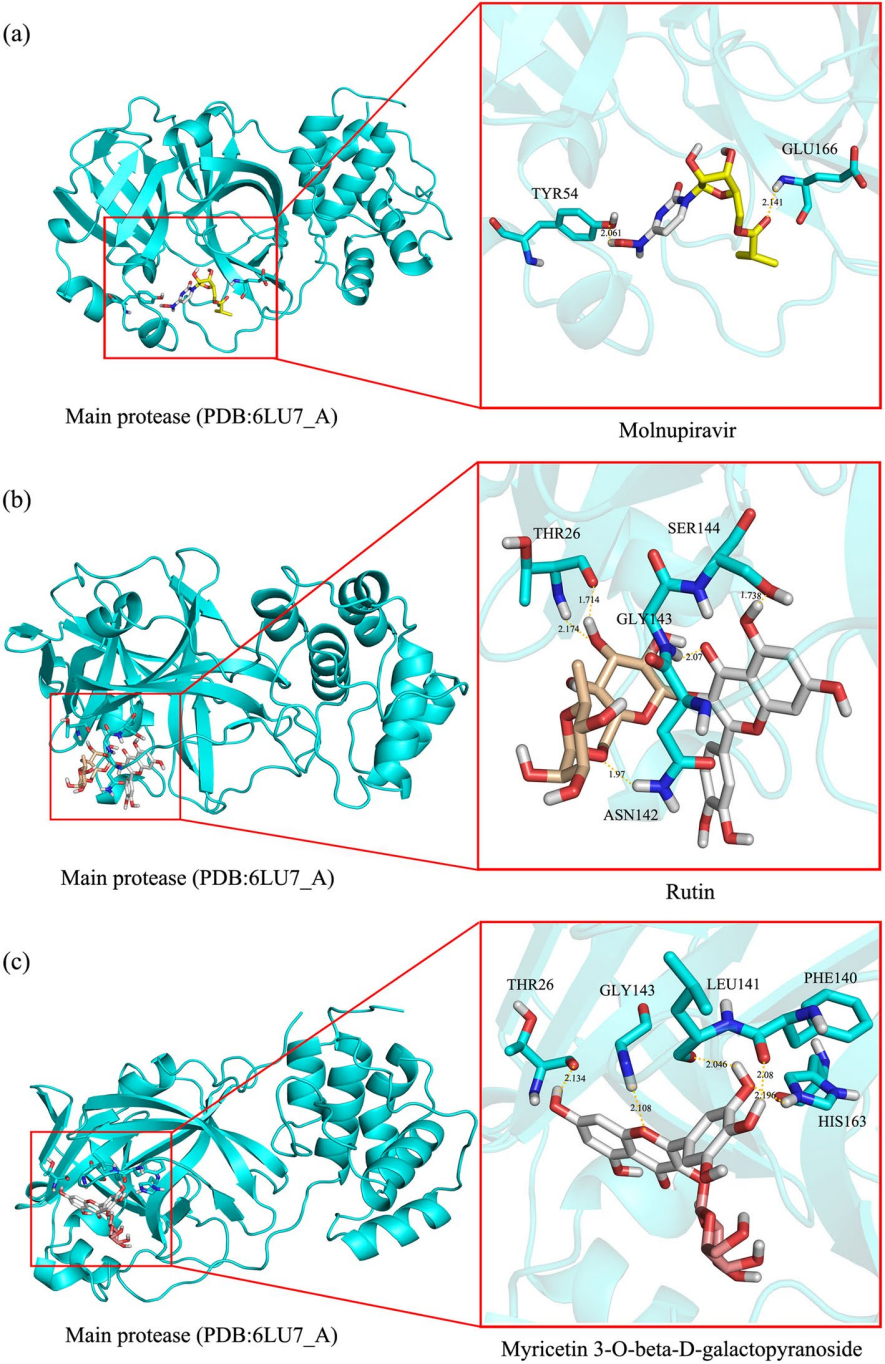
Three-dimensional spaces of molecular docking of principal bioactive compounds with the active site of SARS-CoV-2 main protease (M^pro^). The zoomed-in view of their interaction patterns is presented at the right panels. **a**, **b**, and **c** represent the molecular interaction patterns of molnupiravir, rutin, and myricetin 3-*O*-beta-D-galactoside, respectively, with amino acid residues of M^pro^. The cyan ribbon represents the 3D structure of M^pro^ (PDB code: 6lu7_A). The structures are colored as follows: cyan for carbon in M^pro^, yellow for carbon in molnupiravir, wheat for carbon in rutin, deep-salmon for carbon in myricetin-3-*O*-beta-D-galactoside, gray for hydrogen, blue for nitrogen, and red for oxygen. The interactions of the binding residues from M^pro^ are marked according to the sequence numbering of M^pro^. Hydrogen bonds are shown in yellow dashed lines. Distances are given in Å

The docking analysis showed that these flavonoids and their conjugates efficiently bind by forming hydrogen bonds at the active site of main protease the COVID-19 virus. The 3D binding interactions and positioning within the binding pocket of the M^pro^ of SARS-CoV-2 with molnupiravir, rutin, and myricetin 3-*O*-β-D-galactopyranoside are illustrated in Fig. 4 as examples. Molnupiravir formed two hydrogen bonds to the active site of M^pro^ at Thr-54 and Glu-166 (Fig. 4a). Rutin established five hydrogen bonds with amino acids at the active site of the M^pro^, including Thr-26 (1.714 Å and 2.174 Å), Asn-142 (1.97 Å), Gly-143 (2.07 Å), and Ser-144 (1.738) (Fig. 4b). Myricetin-3-*O*-beta-D-galactoside also effectively bound to the active site by forming five hydrogen bonds with amino acids at the active site of M^pro^, specifically Thr-26 (2.134 Å), Phe-140 (2.08 Å), Leu-141 (2.046 Å), Gly-143 (2.108 Å), and His-163 (2.196 Å) (Fig. 4c).

#### Stability of the docked complexes

**Fig. 5:**
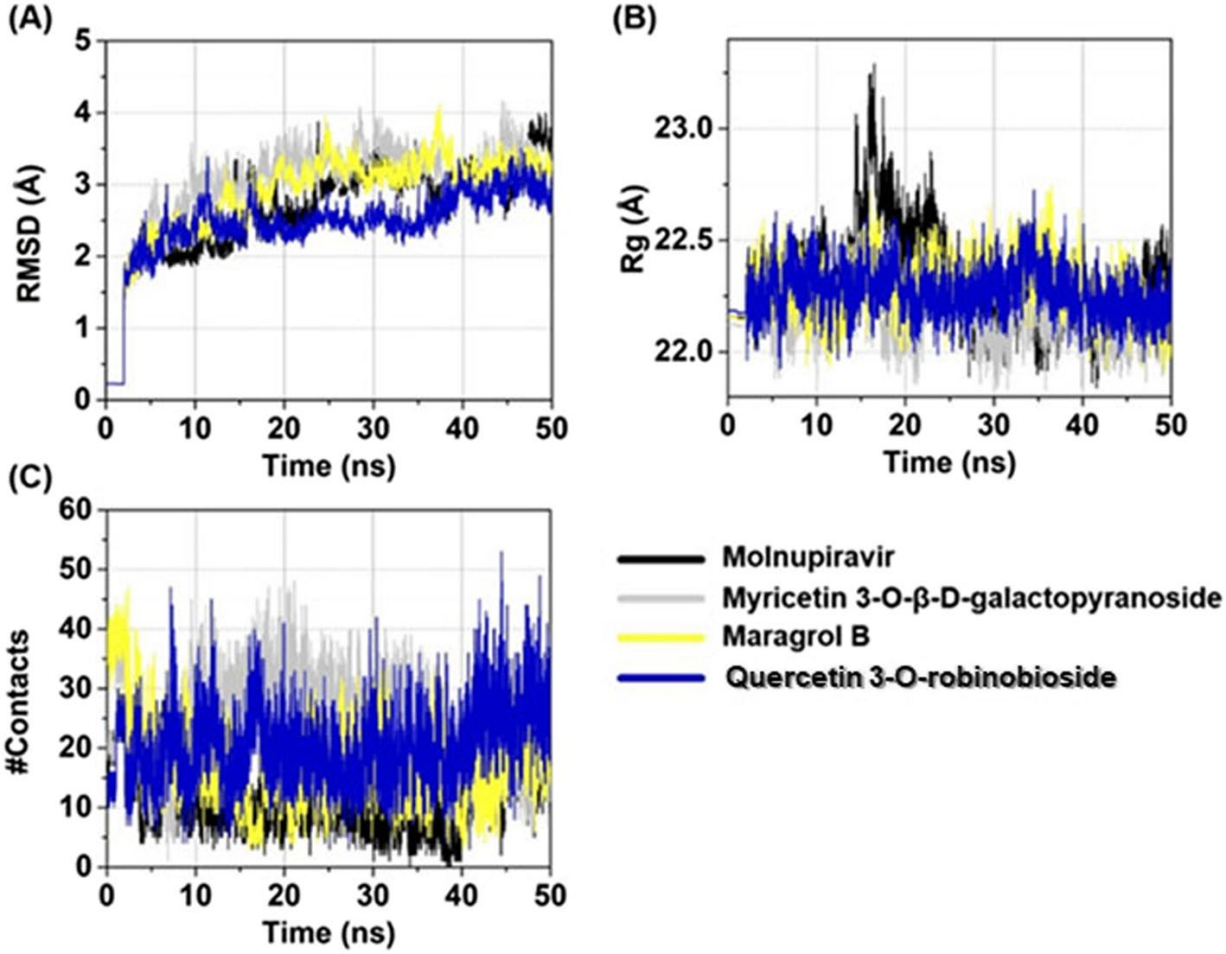
Time evolution of (**A**) RMSD, (**B**) Rg, and (**C**) #Contacts of molnupiravir, myricetin 3-*O*-β-D-galactopyranoside, maragrol B, and quercetin 3-*O*-robinobioside in complexes with the main protease of SARS-CoV-2

The stability in an aqueous environment of the top three docked compounds—myricetin 3-*O*-β-D-galactopyranoside, maragrol B, and quercetin 3-*O*-robinobioside including molnupiravir in complexes with M^pro^, was evaluated by calculating the all-atom RMSD, Rg for protein–ligand complexes, and #Contacts within 3.5 Å of the ligand over 50-ns simulation time. As shown in Fig. 5, overall, the simulated models revealed stable dynamics after 30 ns. The RMSD values of all systems continuously increased during the first 20 ns, but afterward, all complexes achieved stability by showing low fluctuations. The average RMSD values after 30 ns until the end of the simulation time of complexes were observed to be ~ 3.2 ± 0.04 Å, except for the quercetin 3-*O*-robinobioside system (~ 2.8 ± 0.3 Å) (Fig. 5A). The Rg for protein–ligand complex was further computed to describe their structural compactness. All three flavonoid models showed average Rg values of ~ 22 ± 0.1 Å (calculated from the last 20 ns of simulation), suggesting stable protein–ligand binding complexes. The high fluctuation of Rg was observed in the molnupiravir system at ~ 15–20 ns; however, the system seemed to tune back to the unfolding stage afterward (Fig. 5B). Notably, the average #Contacts from 30–50 ns for these three flavonoid glycoside models (19 ± 6, 16 ± 6 and 22 ± 7 for myricetin 3-*O*-β-D-galactopyranoside, maragrol B, and quercetin 3-*O*-robinobioside, respectively) were higher than those of the molnupiravir model (11 ± 6) (Fig. 5C).

## Discussion

Due to a diverse phytochemical content in medicinal plants, it still garnered the interest of researchers in searching for potential candidates for combatting SARS-COV-2. Phytochemicals display promising therapeutic efficacy in the management of COVID-19 because they possess not only antiviral activity but also antioxidant, anti-inflammatory, antibacterial, and immunomodulatory activities [12]. Besides the direct virucidal action, those properties could play a significant role in reducing the severity of COVID-19 caused by hyperinflammatory and dysregulated immune responses [13]. In this study, we conducted bioassay-guided screening for potential therapeutic medicinal plant extract as an anti-SARS-CoV-2 agent through 22 CEs of medicinal plants, based primarily on in vitro anti-SARS-CoV-2 efficacy in Vero E6 cells in two modes of antiviral mechanism—pre-entry phase (as virucidal efficacy) and post-infectious phase (as viral replication inhibition efficacy). The potential CEs underwent liquid–liquid fractionation using a water/ethyl acetate solvent system to enhance anti-SARS-CoV-2 efficacy. This led to the formation of a WF (containing highly polar compounds cluster) and an EF (containing mid-to-nonpolar compounds cluster), based on polarity of the solvent used [73]. Up to this point of our study, this fractionation technique provided the phytocompound cluster with the most outstanding anti-SARS-CoV-2 activity, which was WF-MLCE (S2-WF). WF-MLCE showed significantly higher antiviral potency than the CE and EF of MLs. According to CC_50_ (> 0.7 mg/mL) and IC_50_ (< 15.6 μg/mL) of WF-MLCE, this promising ML extract also tended to be more safe and more efficient in combating SARS-CoV-2 in comparison with *Andrographis paniculata* extract (Fig. 1 and 2, respectively). AP extract had been studied at the clinical trial level for COVID-19 patient treatment, as previously mentioned. The study by Sa-ngiamsuntorn et al. (2021) in Vero E6 cells illustrated its anti-SARS-CoV-2 activity with an IC_50_ of 68 μg/mL and a CC_50_ of > 0.1 mg/mL [20]. This WF-MLCE showed promising antiviral efficacy, which met our aim of ≥ 99.9% antiviral efficacy (LRV of ≥ 3) (Table 2). WF-MLCE was then underlined as the most promising candidate. In addition, the superior anti-SARS-CoV-2 efficacy of WF-MLCE over molnupiravir and other extracts also highlights the success of employing water/ethyl acetate fractionation to MLCE.

After fractionation, WF-MLCE yielded ~ 80% and EF-MLCE yielded ~ 20% in total content of MLCE (data not shown). Together with the result of evaluating the antiviral lead compounds in WF-MLCE using FTIR (Fig. 3) and LC–HRMS (Tables 3 and 4, and Table S5-S7), we strongly provided compelling evidence that flavonoids and phenolic acids were the principal constituents of WF-MLCE. Rutin was found to be the most abundant among the flavonoids and other polyphenolics. Overall, these polyphenolics are suggested to be the key role in contributing the most outstanding anti-SARS-CoV-2 efficacy of WF-MLCE. Our WF-MLCE phytochemical profile corresponded with the investigation of metabolites of MLs by Guo et al. (2023), which indicated a high concentration of polyphenolics, especially flavonoids and flavonoid glycosides; where the dominant part of metabolites encounters more polarity characteristics [74]. Flavonoids such as quercetin, myricetin, and rutin have attracted attention in many aspects of the study of their potential for anti-SARS-CoV-2. The special structural configuration of flavonoids, which comprises of two benzene rings (A and B) joined by a pyrene ring (C) with varying degrees of hydroxylation, is the essential key to their biological activity and their therapeutic applications. Previous computational studies reported on the list of flavonoids that exhibit viral inhibition by interacting with SARS-CoV-2 [75–77].

A computational approach has been employed to identify potential SARS-CoV-2 inhibitors from the collection of repurposed drugs and chosen phytocompounds. The virtual screening using in silico molecular docking and molecular dynamics simulation of the interactions between bioactive compounds and the target viral protein is effective in terms of time and cost savings [78]. The M^pro^ is a popular target for in silico studies for drug development due to its high conservation level and role as a crucial enzyme involved in viral replication. Thus, besides in vitro biochemical assays of the chosen plant extracts, combination the molecular docking and MD simulation studies of phytoconstituents against M^pro^ could effectively shortlist lead candidates [26, 27, 43, 79–82]. Abian et al. (2020) used molecular docking against M^pro^ to undertake a virtual investigation of the anti-SARS-CoV-2 characteristics of 150 compounds. Quercetin showed great promise; however, its limited bioavailability and water solubility make it difficult to utilize as a supplemental medication; however, its limited water solubility and bioavailability possibly pose challenges for its use as a complementary drug [83]. Nguyen et al. (2021) underlined that the ability of flavonoids in combating SARS-CoV-2 M^pro^ seemed favorably structurally dependent. As an M^pro^ inhibitor, they found that myricetin was the most effective flavonol, followed by quercetin, rutin, and kaempferol. They suggested that the greater M^pro^ inhibitory power of myricetin was due to the higher number of substituted hydroxyl groups in the B-ring structure. The result from kaempferol, which was the least effective inhibitor, also emphasized the hypothesis where there is only one substituted-OH at C4′ of the B-ring in its molecule. The study also indicated that a replacement of OH with a sugar moiety could cause an adverse inhibitory effect for rutin (quercetin-3-O-rutinose), compared to quercetin [84]. The larger space taken up by the structural rearrangement of the quercetin-glycosylated form hampers the binding between M^pro^ and rutin. In contrast, Rizzuti et al. (2021) found that rutin effectively fits within the catalytic pocket of M^pro^. The docking analysis and MD simulation showed that the sugar moiety did not interfere with the binding of rutin to the M^pro^ active site. They stated that the rutinoside sugar moiety contributes to rutin's greater solubility (238% bioavailability compared to quercetin), therefore rutin is more accessible to the active site of 3CL^pro^ (M^pro^) and interacts with the catalytic dyad (His-41/Cys-145) [85]. The reports from many studies supported the high potency of rutin toward SARS-CoV-2 M^pro^ [86–88].

Using in silico molecular docking to determine lead prospective SARS-CoV-2 inhibitors of WF-MLCE against Mpro of SARS-CoV-2 (Table 5), we discovered a number of intriguing candidates based on a comparison of the docked score with reference drugs. Overall, molecular docking and MD result most likely confirmed the benefit of a sugar moiety in a flavonoid molecule, which led to a stronger ability to interact with the active site pocket of the M^pro^. The docking scores of sugar-conjugated flavonoids mostly showed lower binding energy and Ki than those of their flavonoid aglycones, suggesting a higher potential for interaction with M^pro^. To further elaborate, the top three sugar-conjugated flavonoids, myricetin-3-*O*-D-galactopyranoside, kaempferol-3-*O*-rutinoside-7-*O*-rhamnoside (maragrol B), and quercetin-3 *O*-robinobioside, including rutin, all showed significantly lower binding energies than their aglycone forms, i.e., myricetin, kaempferol, and quercetin, respectively. Several other flavonoid-sugar conjugates also had strongly caught our attention, as indicated by their binding energies with SARS-CoV-2 M^pro^ lower than − 7 kcal/mol and inhibition constants lower than 4 μM (Table 5). In addition, structural stability of the most three docking score compounds complexed with the SARS-CoV-2 M^pro^ evaluated using MD simulation in the explicit solvent (water in our case) supported the potential of these compounds as antiviral leads in MLE. Each docked complex demonstrated favorable stability across all calculated parameters. The low fluctuation in RMSD and Rg of each simulated docked complex system after 30 ns until the end of the simulation suggested that the docked complex formed stable bonds throughout the simulation period [26, 89, 90]. Considering the binding affinities against SARS-CoV-2 M^pro^ and the stability of the three-flavonoid glycoside-protein complexes, we strongly highlighted myricetin 3-*O*-β-D-galactopyranoside, maragrol B, and bioquercetin as promising anti-SAR-CoV-2 lead compounds of WF-MLCE.

Considering the potent virucidal efficacy of F1, two chlorogenic acids prevalently found trans-5-O-caffeoylquinic acid and 4-O-caffeoylquinic acid garnered our interest due to their broad-spectrum antiviral activities, including against the human immunodeficiency virus (HIV), and their potent antioxidant properties. They have also demonstrated notable inhibitory activity against the hepatitis B virus by blocking viral DNA synthesis in HepG2.2.15 cells [91]. However, based on our in silico docking analysis, the anticipated anti-SARS-CoV-2 mechanism involving the M^pro^ active sites appeared not to be the case for these two compounds. They did not display the prominently effective binding energy compared to molnupiravir. As a result, if these chlorogenic acids play a key role in the anti-SARS-CoV-2 efficacy of WF-MLCE, their mechanism of action is unlikely to interact with SARS-CoV-2 M^pro^.

To the best of our knowledge, myricetin 3-*O*-β-D-galactopyranoside, maragrol B, and quercetin 3-O-robinobioside have never been studied for either virtual interactions with SARS-CoV-2 proteins or in vitro and in vivo anti-SARS-CoV-2 experiments. To some degree, we suggested the sugar adduct in the flavonoid conjugate molecule is one of the pivotal structure-related parts that positively impact the anti-SARS-CoV-2 efficiency of this MLE, not only the polyhydroxy groups. The sugar moiety may support the overall solubility of the whole fraction (and may be of an entire WF-MLCE). Therefore, it impacts the more accessible extract toward the active site of the target viral protein. However, it is worth noting that, despite the higher solubility of glycosylated conjugate flavonoids compared to the flavonoid aglycone itself, these conjugates have a larger molecular size. Therefore, the molecule’s structure might pose some challenges regarding drug delivery. Taken all together, WF-MLCE showed promising characteristics as an anti-SARS-CoV-2 natural extract candidate. Further comprehensive studies are recommended to assess in vivo toxicity and anti-SARS-CoV-2 activities and to gain a deeper understanding of its mechanism of action. Additionally, it is crucial to investigate the development of an efficient and consistent extraction process because WF-MLCE is not a pure compound extract but rather a cluster of various compounds. The standardization of the extract should also be concurrently obtained in order to produce a qualified extract for clinical usage purposes.

Mulberry (*Morus* spp.) is widely grown in several countries, including India, Korea, Japan China, and Thailand. Mulberry leaves, often used to feed silkworms in sericulture, have been commercially marketed as a form of special tea or drink in many Asian nations [74]. Given that, MLs offer an affordable and readily accessible source of an active anti-SARS-CoV-2 agent. The advancement of clinical and therapeutic studies could potentially result in its widespread adoption as an alternative or complementary agent. Such an approach could not only prove valuable in managing COVID-19 but also have implications for other human coronavirus diseases, both those currently affecting us and any potential future epidemics. The exploration of MLs as a potential treatment option shows promise for viral diseases and warrants further investigation and exploration.

## Conclusions

This study explored the potential medicinal plant extract based on an in vitro anti-SARS-CoV-2 assay in the pre-entry and post-infectious phases. WF-MLCE was underlined as the most promising anti-SARS-CoV-2 candidate, while observing low cytotoxicity. The evaluation of antiviral lead compounds in WF-MLCE found the extract to be a rich source of polyphenolics, especially flavonoids and conjugates. To the best of our knowledge, this study demonstrates for the first time that the three flavonoid glycosides (myricetin 3-*O*-β-D-galactopyranoside, maragrol B, and quercetin 3-O-robinobioside) could be highlighted as promising antiviral lead molecules of WF-MLCE through molecular docking and MD simulation of the docked complex stability targeting SARS-CoV-2 main protease. Considering all the findings, the WF of ethanolic-aqueous MLCE warrants recognition as a potential alternative natural agent against SARS-CoV-2.

### Supplementary Information

Supplementary Material 1.

Supplementary Material 2.

## Data Availability

All data generated or analyzed during this study are included in this published article [and its additional file].

## Abbreviations

SARS-CoV-2: Severe Acute Respiratory Syndrome Coronavirus 2
COVID-19: Coronavirus Disease 2019
PEDV: Porcine Epidemic Diarrhea Virus
LC-HRMS: Liquid Chromatography-High Resolution Mass Spectrometry
MTT Assay: 3,4,5-Dimethylthiazol-2-yl)-2,5-diphenyltetrazolium bromide assay
MNTC: Maximum Non-Cytotoxic Concentration
TCID_50_: 50% Tissue Culture Infective Dose
LRV: Log Reduction Value
