# Supplementary material for: Exploring the therapeutic potential of Thai medicinal plants: in vitro screening and in silico docking of phytoconstituents for novel anti-SARS-CoV-2 agents
Source: BMC Complement Med Ther. 2024 Jul 19;24:274. doi: 10.1186/s12906-024-04586-z (PMC11264683; doi:10.1186/s12906-024-04586-z)
Supplement: Supplementary file 1 — Supplementary Material 1. [file 12906_2024_4586_MOESM1_ESM.docx]

**Additional File 1.doc**

**Supplementary Methods**

**Table S1** Plants materials

**Fig. S1** A Schematic diagram with an overview of the study design and the main procedures.

**SI 1**. Preparation of PEDV

**SI 2.** Anti-viral activity of crude extracts against PEDV as a surrogate

**SI 3**. Calculation of anti-SARS-CoV-2 efficacy and percentage of viral reduction

**SI 4**. Estimation of polyphenols and antioxidant assessments

**Table S1** Information of plants utilized in this research includes their assigned voucher specimen numbers (TTM no.), used plant parts for extraction and harvesting areas in Thailand.

| **Sample Code** | **Scientific name  [part of use]** | **Common Thai name** | **TTM no.** | **Harvesting area** |
| --- | --- | --- | --- | --- |
| S1 | *Colubrina asiatica* (L.) Brongn. [leaves] | Khan song | 0005480 | Nakhonratchasima |
| S2 | *Morus alba* Linn.  [leaves] | Mon | 0005481 | Nakhonratchasima |
| S3 | *Gynostemma pentaphyllum* (Thunb) Makino  [leaves] | Jiaogulan | 0005482 | Chiang Mai |
| S4 | *Artemisia annua* L.  [leaves] | Kot chula lampha | 0005483 | Kanchanaburi |
| S5 | *Centella asiatica* (L.)  [leaves] | Bua bok | 0005484 | Mahasarakham |
| S6 | *Justicia gendarussa* Burm.f. [leaves and stems] | San phra mon | 1000745 | Sing Buri |
| S7 | *Helicteres isora* L.  [fruits] | Po bid | 0005485 | Nakhonpathom |
| S8 | *Phyllanthus niruri* L.  [leaves, fruits, and stems] | Lok tai bai | 0005486 | Burirum |
| S9 | *Orthosiphon aristatus*  [leaves and stem] | Ya nuat maew | 0005487 | Nakhonpathom |
| S10 | *Oldenlandia corymbosa* L. [leaves, flowers, and stems] | Ya lin ngu | 0005488 | Chai Nat |
| S11 | *Garcinia atroviridis*  [leaves] | Som khaek | 0005489 | Nakhonpathom |
| S12 | *Canna indica* L.  [stems] | Phutha raksa | 1000746 | Sing Buri |

**Table S1** (Continued)

| **Sample Code** | **Scientific name [part of use]** | **Common Thai name** | **TTM no.** | **Harvesting area** |
| --- | --- | --- | --- | --- |
| S13 | *Euphorbia thymifolia* L.  [leaves and stems] | Nam nom ratchasi lek | 0005490 | Nakhonratchasima |
| S14 | ‎*Acanthus ebracteatus* Vahl [leaves and stems] | Ngueak pla mo | 1000747 | Sing Buri |
| S15 | *Clinacanthus nutans* (Burm.f) Lindua  [leaves and stems] | Phaya plong thong | 1000748 | Sing Buri |
| S16 | *Jatropha integerrima*  [flowes] | Pattawia | 0005491 | Nakhonratchasima |
| S17 | *Jatropha integerrima*  [leaves] | Pattawia | 0005491 | Nakhonratchasima |
| S18 | *Jatropha integerrima*  [stems] | Pattawia | 0005491 | Nakhonratchasima |
| S19 | *Punica granatum* L.  [leaves] | Thapthim | 0005492 | Nakhonratchasima |
| S20 | *Momordica charantia* L. [fruits] | Mara kee nok | 0005493 | Nakhonratchasima |
| S21 | *Hymenocallis littoralis*(Jacq.) Salisb*.* [Root] | Plub plueng tin pade | 0005494 | Nakhonratchasima |
| S22 | *Hymenocallis littoralis*(Jacq.) Salisb [Leaves] | Plub plueng tin pade | 0005494 | Nakhonratchasima |

**
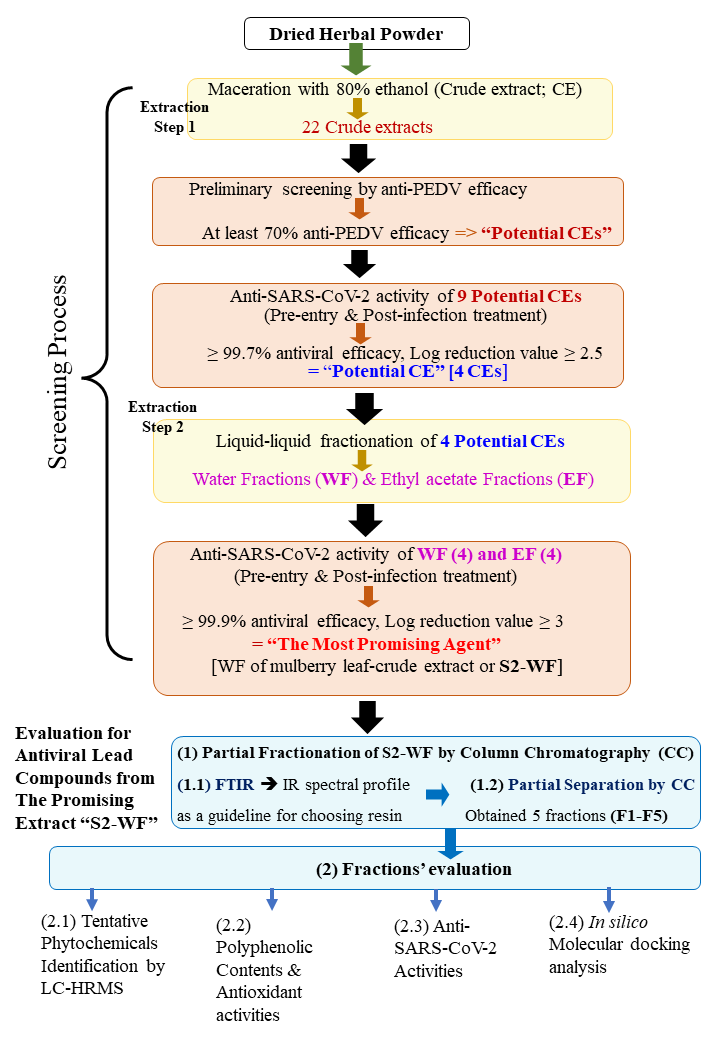
**

**Fig. S1** A Schematic diagram with an overview of the study design and the main procedures.

**SI 1. Preparation of PEDV**

PEDV was propagated in Vero cell by culturing in serum-free DMEM supplemented with 10 μg/μL Trypsin, TPCK Treated (Trypsin 1:250, Gibco) and 0.3% Tryptose Phosphate Broth (TPB, Sigma-Aldrich) (infection medium). Once cytopathic effect (CPE) reached 70–80% (about 18–24 h post-infection), the cells were subjected to three freeze-thaw cycles. The supernatant was collected, aliquoted, and stored at −80 °C until further use. Virus stock was quantified for viral titer using plaque assay following Wang *et al.* (2005)*.* Briefly, PEDV stock was diluted in 10-fold serial dilution concentrations and then inoculated into Vero cells (CCL-81) followed by incubation at 37 °C for 4-5 days to allow the cell infection and form the plaques. The cells were fixed with 0.5% agar, stained by crystal violet, and the plaque number was counted. Virus titer was quantified and expressed in plaque forming unit per mL (pfu/mL).

**SI 2. Anti-viral activity of crude extracts against PEDV as a surrogate**

The first screening step of potential crude extracts against SARS-CoV-2 of all 22 crude extracts was performed using PEDV as a surrogate. The single dose concentration of individual crude extract (their particular safe concentration obtained from MTT assay on Vero cells) was applied for the virucidal activity against PEDV. The efficacy of the crude extract in reducing the amount of porcine coronavirus was evaluated using the plaque reduction neutralization test (PRNT) following Wang *et al.* (2005). PEDV were incubated with individual crude extracts for an exposure period of 5 min at 37 °C. The sample was collected and detected for the remaining infectious PEDV in the Vero cell (CCL-81). The remaining viral solution was serially diluted in 10-fold dilutions, inoculated into the Vero cells, and incubated at 37 °C for 3 days, allowing the infected cells to form the plaques. The cells were fixed, stained with crystal violet, and the plaque number was counted. The amount of virus was quantified and expressed in plaque-forming unit per mL (pfu/mL). The virucidal activity against PEDV was evaluated by the reduction of plaque formation in comparison with the control (untreated-PEDV) and reported the efficacy as a percentage of viral reduction. The crude extract showed at least 70% efficacy and was subjected to anti-SARS-CoV-2 activity studies.

**SI 3. Calculation of anti-SARS-CoV-2 Efficacy and percentage of viral reduction**

In the present study, the anti-SARS-CoV-2 activity of tested extract as related to the viral reduction was expressed as efficacy (percentage of viral reduction) and Log reduction value (LRV). The relation of the 2 values was as follows.

| **Number of viruses (TCID50/ml)** | **Log reduction** | **Efficacy/**  **% viral reduction** |
| --- | --- | --- |
| 100,000 (10^5^) | 0 log (Log0) | 0% |
| 10,000 (10^4^) | 1 log (Log1) | 90% |
| 1,000 (10^3^) | 2 log (Log2) | 99% |
| 100 (10^2^) | 3 log (Log3) | 99.9% |
| 10 (10) | 4 log (Log4) | 99.99% |
| 1 (1) | 5 log (Log5) | 99.999% |

**Equations:**

**Efficacy/% viral reduction** $=\frac{\left( A-B \right)x 100}{A}$

Log Reduction Value $=\log_{10} A-\log_{10} B$

where A = The starting viral titer subjected to the test

B = The remaining viral number after testing

**Example of calculation**

The starting viral number for the pre-entry study was 100,000 (10^5^) TCID50/mL. After testing, the remaining viral number was 100 (10^2^) TCID50/mL.

| **Efficacy/**Percent Reduction **=** $\frac{\left( 100,0000-100 \right)x 100}{100,000}$ |
| --- |
| **=** 99.9% |
|  |
| Log Reduction $=\log_{10} 100,000-\log_{10} 100$ |
| **=** 3 Log reduction |

**SI 4. Estimation of polyphenols and antioxidant assessments**

**Total phenolic content (TPC)** was measured using Folin-Ciocalteu reagent using gallic acid (GA) as a standard phenolic compound. A 25 μL of Folin-Ciocalteu reagent solution (1:3 diluted with water) was mixed with 25 μL sample and 200 μL of water in a 96-well plate. After incubation at room temperature for 5 min, 25 μL of 10% (*w/v*) Na_2_CO_3_ was added, followed by incubation with light protection for 60 min. The absorbance of the mixture was measured at 765 nm using a microplate reader. TPC in samples was calculated from a calibration curve obtained from GA (concentration range of 0-250 μg/mL) and expressed as mg gallic acid equivalents (GAE) per gram of dried sample weight (mg GAE/g extract).

**Total flavonoid content (TFC)** was determined by the aluminum chloride method using quercetin (Q) as a flavonoid standard. The reaction was performed by mixing 25 μL of the sample with 100 μL of water and 10 μL of 10 g/L NaNO_2_ in a 96-well plate, followed by incubation at room temperature for 5 min. Then, 15 μL of 100 g/L AlCl_3_ was added, and the incubation was continued for 6 min prior to adding 50 (L) of water and 50 μL of 1 M NaOH. The plate was shaken for 30 sec before completing the absorbance measurement at 415 nm using a microplate reader. TFC was quantified using a calibration curve constructed from quercetin (50-1,000 μg/mL) and reported as mg of quercetin equivalents per g of dried extract (mg QE/g extract).

**DPPH** radical (DPPH^⚫^) scavenging activity was measured using Trolox as the antioxidant standard. The extract samples or Trolox were diluted to the required concentration with 70% (*v/v*) methanol before subjecting to the reaction. Briefly, 50 μL of the sample was mixed with 250 μL of 0.2 mM DPPH in methanol in a 96-well plate. The reaction mixture was placed in the dark for 30 min before the absorbance reading was commenced at 517 nm using a microplate reader. DPPH^⚫^ radical scavenging capacity of the samples was estimated from a standard curve of Trolox (10-200 μM). The result was expressed as micromoles of Trolox equivalents per gram of dried extract (μmol TE/g extract). The extract concentration (mg/mL) at which scavenged 50% of DPPH^⚫^ (EC50) was also estimated.

**ABTS** radical (ABTS^+⚫^) scavenging activity was conducted by ABTS radical cation decolorization assay using Trolox as the antioxidant standard. The ABTS^+⚫^ were firstly produced by mixing 5 mL of 7 mM of ABTS solution with 175 μL of 140 mM potassium persulfate, then the mixture was volume up to 10 mL by adding water, then stood overnight (≥ 16 h) at room temperature in the dark to ensure the radical production completed. Subsequently, the ABTS^+^ solution was diluted with sodium phosphate buffer (66 mM, pH 3.6) to gain its absorbance of 0.7±0.05 at 734 nm before using as the working ABTS+ solution for the reaction. To conduct the reaction, 10 μL of the sample was reacted with 290 μL of working ABTS+ solution and then incubated in the dark for 6 min before reading the absorbance at 734 nm. The antioxidant capacity was reported as micromoles of Trolox equivalents per gram of dried extract (μmol TE/g extract). The extract concentration (mg/mL) at which eliminated 50% ABTS+ radicals (EC50) was also estimated.

**FRAP** assay was estimated based on the ability of the sample to reduce Fe^3+^ (Fe^3+^-TPTZ complex) to Fe^2+^ (Fe^2+^-TPTZ complex). FRAP reagent was prepared by mixing 300 mM of sodium acetate with 10 mM TPTZ (freshly prepared in 40 mM HCl) and the freshly prepared 20 mM FeCl_3_ in the volume ratio of 10:1. The FRAP reagent was placed at 37 °C throughout the assay. The assay was performed by reacting 20 μL of the sample with 280 μL of FRAP reagent at 37 °C for 10 min before acquiring the absorbance at 593 nm. The quantitation of the FRAP result of the sample was based on a standard curve of Trolox (0-1000 μM) and reported as μmol TE/g extract.
