# Supplementary material for: Exploring the therapeutic potential of Thai medicinal plants: in vitro screening and in silico docking of phytoconstituents for novel anti-SARS-CoV-2 agents
Source: BMC Complement Med Ther. 2024 Jul 19;24:274. doi: 10.1186/s12906-024-04586-z (PMC11264683; doi:10.1186/s12906-024-04586-z)
Supplement: Supplementary file 2 — Supplementary Material 2. [file 12906_2024_4586_MOESM2_ESM.docx]

**Additional File 2.doc**

**Table S2** Anti-PEDV efficacy as %virucidal of twenty-two crude extracts.

**Fig. S2** Cytotoxicity of the nine crude extracts (S1-S8) with CC_50_, evaluated over Vero E6 cells.

**Table S3** Anti-SARS-CoV-2 efficacy of the nine crude extracts, evaluated in pre-entry and postinfection treatment study.

**Fig. S3** Fraction collection of WF-MLCE (S2-WF) obtained from column chromatography by Sephadex LH-20 resin.

**Table S4** FTIR spectral band assignment for water fraction of mulberry leaf-crude extract.

**Table S5** LC–HRMS analysis of tentative phytochemical contents of Fraction 2 fractionated from WF-MLCE (S2-WF).

**Table S6** LC–HRMS analysis of tentative phytochemical contents of Fraction 3 fractionated from WF-MLCE (S2-WF).

**Table S7** LC–HRMS analysis of tentative phytochemical contents of Fraction 4 fractionated from WF-MLCE (S2-WF).

**Fig. S4** Cytotoxicity of five fractions obtained from column chromatography of WF-MLCE, evaluated over Vero E6 cells.

**Table S8** Anti-SARS-CoV-2 efficacy of the five fractions obtained from column chromatography of WF-MLCE (S2-WF).

**Table S9** Polyphenol contents and antioxidant activities of fractions obtained from column chromatography of WF-MLCE.

**Table S2** Anti-PEDV efficacy (%virucidal) of 22 crude extracts with 5 minute of contact time.

| **Sample Code** | **Thai common name** | **Tested conc. (μg/mL)** | **Efficacy (%)** |
| --- | --- | --- | --- |
| S1 | Khan song | 1000.0 | 89 |
| S2 | Mon | 125.0 | 89 |
| S3 | Jiaogulan | 500.0 | 87 |
| S4 | Kot chula lampha | 1000.0 | 91 |
| S5 | Bua bok | 500.0 | 70 |
| S6 | San phra mon | 1000.0 | 70 |
| S7 | Po bid | 125.0 | 70 |
| S8 | Lok tai bai | 125.0 | 95 |
| S9 | Ya nuat maew | 125.0 | 62 |
| S10 | Ya lin ngu | 250.0 | 67 |
| S11 | Som khaek | 1000.0 | 62 |
| S12 | Phutha raksa | 750.0 | 66 |
| S13 | Nam nom ratchasi lek | 62.5 | 54 |
| S14 | Ngueak pla mo | 125.0 | 57 |
| S15 | Phaya plong thong | 500.0 | 64 |
| S16 | Pattawia (flower) | 125.0 | 68 |
| S17 | Pattawia (leaves) | 125.0 | 57 |
| S18 | Pattawia (stem) | 250.0 | 54 |
| S19 | Thapthim (leaves) | 62.5.0 | 50 |
| S20 | Mara kee nok | 125.0 | 62 |
| S21 | Plub plueng tin pade (root) | 62.5 | 61 |
| S22 | Plub plueng tin pade (leaves) | 62.5 | 61 |

**Note:** After the ethanolic evaporation using a vacuum rotary evaporator, S8-CE separated into two parts: a light soluble component (S8-L) and a dark-gummy component (S8-D). During the rapid anti-PEDV screening, these two parts were combined in a 1:1 ratio before testing, including the cytotoxicity test. However, in the subsequent study, these two components were used and tested separately, resulting in nine CEs being evaluated for their anti-SARS-CoV-2 activity.

**
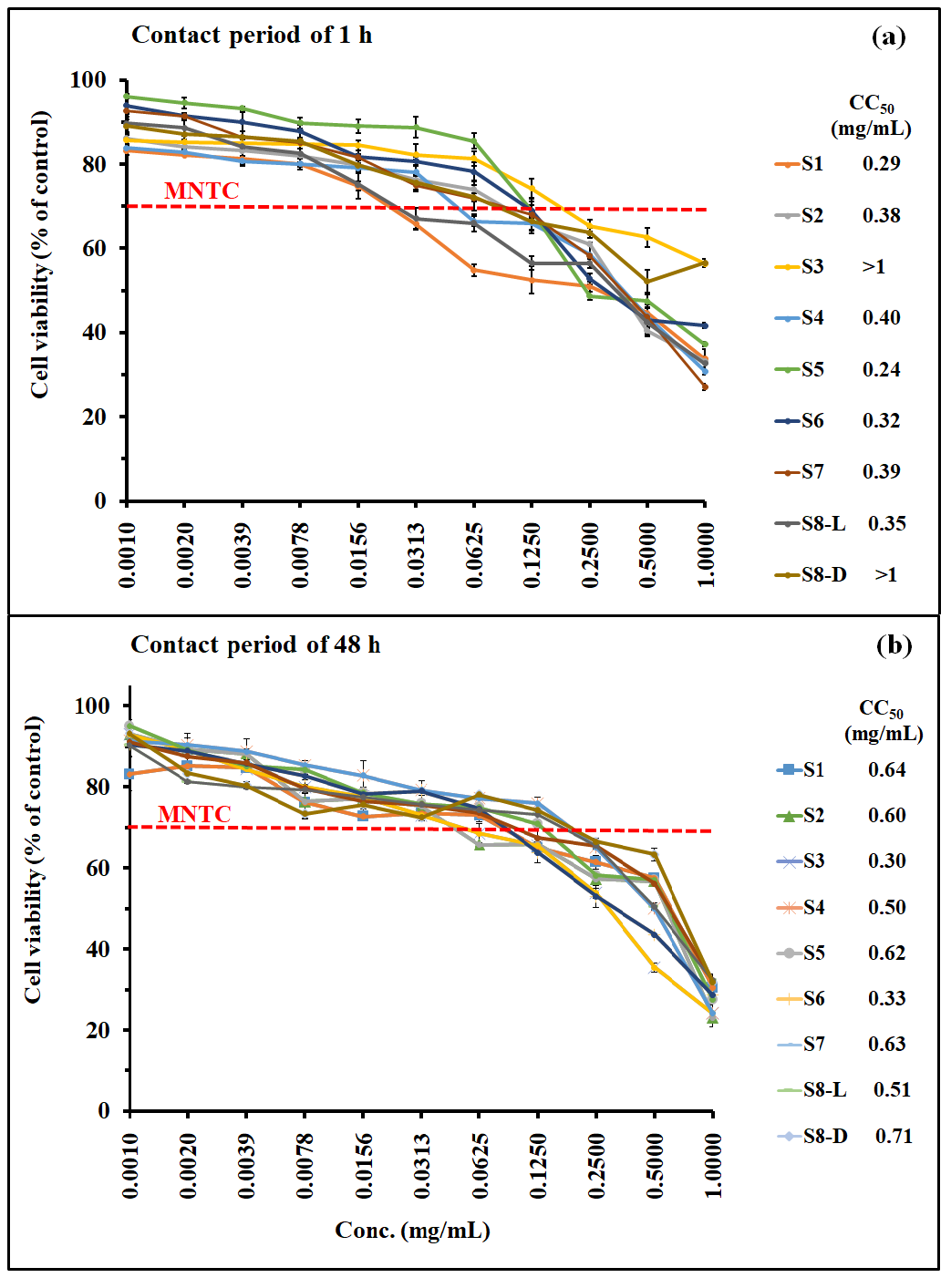
**

**Fig. S2** Cytotoxicity of the nine crude extracts (S1-S8) with CC_50_, evaluated over Vero E6 cells. Results are expressed as % cell viability at various extract concentration (mean ± SD). (a) after 1 h of treatment of crude extracts, and (b) after 48 h of treatment of crude extracts. MNTC-red dot line indicates the minimum non-toxicity concentration (MNTC) with 70% cell viability of individual crude extract.

**Table S3** Anti-SARS-CoV-2 efficacy of the nine crude extracts, evaluated in pre-entry and postinfection treatment study.

| Crude Extract | Pre-entry study | | |  | Postinfection treatment study | | |
| --- | --- | --- | --- | --- | --- | --- | --- |
|  | Conc.  (mg/mL) | Anti-SARS-CoV-2 efficiency | |  | Conc.  (mg/mL) | Anti-SARS-CoV-2 efficiency | |
|  |  | Log Reduction | % Virucidal |  |  | Log Reduction | % Inhibition |
| S1 | 0.0156 | 2.10 ± 0.22 | 99.21 ± 0.26 |  | 0.0625 | 2.52 ± 0.22 | 99.69 ± 0.13 |
|  | 0.0078 | 2.10 ± 0.22 | 99.21 ± 0.26 |  | 0.0313 | 2.35 ± 0.14 | 99.56 ± 0.17 |
|  | 0.0039 | 1.98 ± 0.14 | 98.96 ± 0.32 |  | 0.0156 | 2.27 ± 0.21 | 99.46 ± 0.18 |
| S2 | 0.0625 | 2.10 ± 0.22 | 99.21 ± 0.26 |  | 0.0313 | 2.60 ± 0.21 | 99.75 ± 0.08 |
|  | 0.0313 | 2.02 ± 0.12 | 99.08 ± 0.16 |  | 0.0156 | 2.40 ± 0.30 | 99.54 ± 0.36 |
|  | 0.0156 | 1.98 ± 0.07 | 99.00 ± 0.00 |  | 0.0078 | 2.52 ± 0.44 | 99.62 ± 0.26 |
| S3 | 0.1250 | 2.06 ±0.14 | 99.16 ± 0.18 |  | 0.0313 | 2.52 ± 0.22 | 99.69 ± 0.13 |
|  | 0.0625 | 2.02 ±0.02 | 99.08 ± 0.16 |  | 0.0156 | 2.31 ± 0.38 | 99.45 ± 0.27 |
|  | 0.0313 | 1.94 ±0.15 | 98.88 ± 0.23 |  | 0.0078 | 2.35 ± 0.30 | 99.53 ± 0.24 |
| S4 | 0.0313 | 1.98 ± 0.07 | 99.00 ± 0.00 |  | 0.1250 | 2.10 ± 0.22 | 99.21 ± 0.26 |
|  | 0.0156 | 1.94 ± 0.15 | 98.88 ± 0.23 |  | 0.0625 | 2.02 ± 0.02 | 99.08 ± 0.16 |
|  | 0.0078 | 1.94 ± 0.15 | 98.88 ± 0.23 |  | 0.0313 | 1.94 ± 0.15 | 98.88 ± 0.23 |
| S5 | 0.0625 | 2.06 ± 0.22 | 99.13 ± 0.27 |  | 0.1250 | 2.12 ± 0.14 | 99.37 ± 0.11 |
|  | 0.0313 | 1.98 ± 0.07 | 99.00 ± 0.00 |  | 0.0625 | 1.98 ± 0.07 | 98.96 ± 0.32 |
|  | 0.0156 | 1.98 ± 0.07 | 99.00 ± 0.00 |  | 0.0313 | 1.85 ± 0.12 | 98.65 ± 0.23 |

Results are expressed as mean ± SD of quadruplicate experiments. Log reduction, the reduction of viral titer after experimental treatment in logarithmic function; % Virucidal or % Inhibition, % viral titer reduction compared with the initial viral load. S1- S8, herbal names were listed as described in Table S1.

**Table S3** (Continued)

| Crude Extract | Pre-entry study | | |  | Postinfection treatment study | | |
| --- | --- | --- | --- | --- | --- | --- | --- |
|  | Conc.  (mg/mL) | Anti-SARS-CoV-2 efficacy | |  | Conc.  (mg/mL) | Anti-SARS-CoV-2 efficacy | |
|  |  | Log Reduction | % Virucidal |  |  | Log Reduction | % Inhibition |
| S6 | 0.0625 | 2.10 ± 0.22 | 99.21 ± 0.26 |  | 0.0625 | 2.44 ± 0.07 | 99.65 ± 0.07 |
|  | 0.0313 | 2.06 ± 0.14 | 99.12 ± 0.39 |  | 0.0313 | 2.19 ± 0.14 | 99.37 ± 0.11 |
|  | 0.0156 | 2.02 ± 0.12 | 99.04 ± 0.37 |  | 0.0156 | 1.94 ± 0.07 | 98.88 ± 0.23 |
| S7 | 0.0625 | 2.06 ± 0.22 | 99.13 ± 0.27 |  | 0.0625 | 1.94 ± 0.07 | 98.88 ± 0.23 |
|  | 0.0313 | 2.02 ± 0.17 | 99.04 ± 0.37 |  | 0.0313 | 1.98 ± 0.07 | 99.00 ± 0.00 |
|  | 0.0156 | 1.94 ± 0.07 | 98.88 ± 0.23 |  | 0.0156 | 1.85 ± 0.12 | 98.65 ± 0.23 |
| S8-L  (Light-water soluble) | 0.0156 | 2.27 ± 0.21 | 99.46 ± 0.18 |  | 0.1250 | 2.56 ± 0.30 | 99.68 ± 0.25 |
|  | 0.0078 | 2.35 ± 0.49 | 99.31 ± 0.66 |  | 0.0625 | 2.31 ± 0.38 | 99.45 ± 0.27 |
|  | 0.0039 | 2.27 ± 0.43 | 99.27 ± 0.61 |  | 0.0313 | 2.06 ± 0.14 | 99.16 ± 0.18 |
| S8-D (Dark-gummy) | 0.0625 | 2.56 ± 0.30 | 99.68 ± 0.25 |  | 0.1250 | 2.94 ± 0.07 | 99.89 ± 0.02 |
|  | 0.0313 | 2.44 ± 0.28 | 99.60 ± 0.23 |  | 0.0625 | 2.65 ± 0.25 | 99.77 ± 0.10 |
|  | 0.0156 | 2.31 ± 0.38 | 99.45 ± 0.27 |  | 0.0313 | 2.56 ± 0.22 | 99.73 ± 0.08 |

Results are expressed as mean ± SD of quadruplicate experiments. Log reduction, the reduction of viral titer after experimental treatment in logarithmic function; % Virucidal or % Inhibition, % viral titer reduction compared with the initial viral load. S1- S8, herbal names were listed as described in Table S1.

**Table S4** FTIR spectral band assignment for water fraction of mulberry-leaf crude extract.

| Region | Wave number  (cm^-1^) | Function group  assignment | Possible predicted phytochemical class | Ref. |
| --- | --- | --- | --- | --- |
| 1 | 3500-3000  (broaden) | H-bonded, O-H, N-H | alcohols, polyphenolics, carbohydrates | 40,  56-58 |
| 2 | 3000-2800 | C-H of CH_2_ & CH_3_ | polysaccharides, fatty acids, lipids | 40,  56-58 |
| 3 | 1750-1650 | C=O of carboxylic group | lipids, phenolics | 58 |
|  | 1650-1550 (distinct)  &1500-1455 (small) | C=C-C and C-H of aromatic rings | flavonoids | 58 |
| 4 | 1500-1150 | O-H deformation, C-O, C=C of phenols | flavonoids (flavonols), phenols | 56 |
|  | 1499-1452 | -C-OH and C-H of aromatic rings | Flavonoids (flavones & flavonols), phenolics | 40, 59 |
|  | 1450-1200 | O-H deformation of aromatic rings | flavonoids, phenolics | 59 |
| 5 | 1100-1075 | C-H & C-H out-of-plane of Ring B-flavonoids | flavonoids | 56, 58, 59 |
|  | 1041 (distinct) &  1103 (small) | C-O of aromatic rings,  C-O-C of heterocyclic ring, O-H in-plane | phenolics  saccharides/ carbohydrates | 56, 58, 59 |
|  | 990 (distinct) | C-H out-of-plane of pyranosyl rings | sugars (if appeared with peaks at 1140-1120, suggested the sugar molecules conjugated with aromatic structure) | 56, 58, 59 |
|  | 925 | C-H & C-H out-of-plane of Ring B-flavonoids | flavonoids | 56, 58, 59 |
| 6 | 800-400 | C-C-O deformation,  C-C-C of aromatics,  C-H deformation of polyphenols | flavonoids | 56, 58, 59 |

**
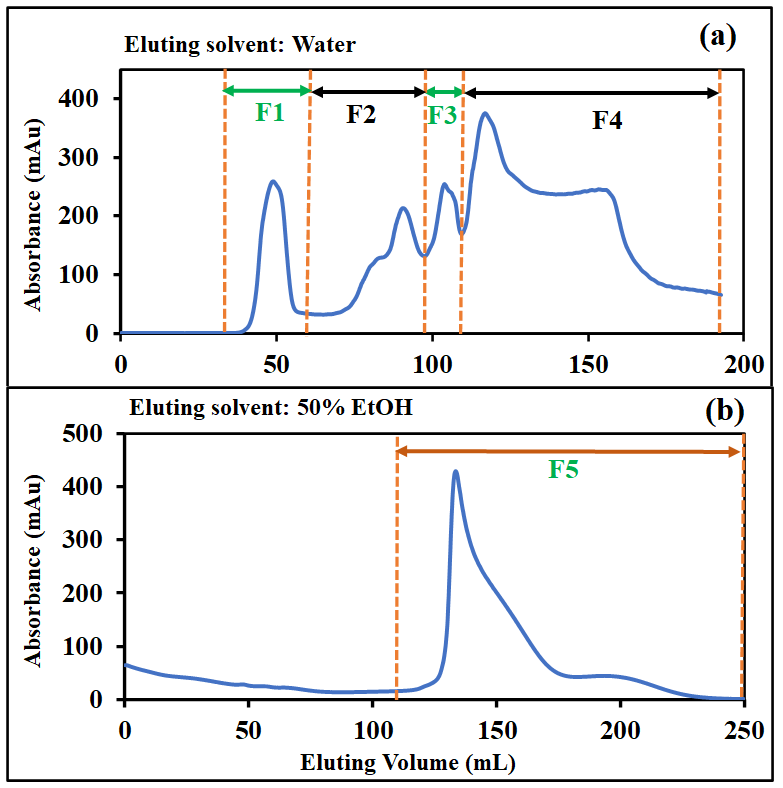
**

**Fig. S3** Fraction collection of WF-MLCE (S2-WF) obtained from column chromatography by Sephadex LH-20 resin. (a) fractions eluted with water, and (b) fractions eluted with 50% ethanolic aqueous.

**Table S5** LC–HRMS analysis of tentative phytochemical contents of Fraction 2 fractionated of WF-MLCE (S2-WF).

| No. | Tentative phytochemical | Formula | Theoretical MW | Reference ion | Average Peak Area (from Most Common Adduct) |
| --- | --- | --- | --- | --- | --- |
| 1 | Trigonelline | C_7_H_7_NO_2_ | 137.04768 | [M+H]^+^ | 3.95E+09 |
| 2 | Υ-Aminobutyric acid (GABA) | C_4_H_9_NO_2_ | 103.06333 | [M+H]^+^ | 1.95E+09 |
| 3 | L-Norleucine | C_6_H_13_NO_2_ | 131.09463 | [M+H]^+^ | 1.06E+09 |
| 4 | L-Glutamic acid | C_5_H_9_NO_4_ | 147.05316 | [M+H]^+^ | 9.28E+08 |
| 5 | Proline | C_5_H_9_NO_2_ | 115.06333 | [M+H]^+^ | 9.05E+08 |
| 6 | Prolylleucine | C_11_H_20_N_2_O_3_ | 228.14739 | [M+H]^+^ | 8.46E+08 |
| 7 | Isoleucine | C_6_H_13_NO_2_ | 131.09463 | [M+H]^+^ | 5.86E+08 |
| 8 | Valine | C_5_H_11_NO_2_ | 117.07898 | [M+H]^+^ | 5.42E+08 |
| 9 | 1-deoxymannojirimycin or 1-deoxynorijimycin | C_6_H_13_NO_4_ | 163.08446 | [M+H]^+^ | 4.70E+08 |
| 10 | Pantothenic acid | C_9_H_17_NO_5_ | 219.11067 | [M-H]^-^ | 4.17E+08 |
| 11 | DL-Carnitine | C_7_H_15_NO_3_ | 161.10519 | [M+H]^+^ | 3.29E+08 |
| 12 | 3-Methoxybenzaldehyde | C_8_H_8_O_2_ | 136.05243 | [M+H]^+^ | 2.66E+08 |
| 13 | D-(-)-Glutamine | C_5_H_10_N_2_O_3_ | 146.06914 | [M+H]^+^ | 2.50E+08 |

**Table S5 (Continued**). Fraction 2

| No. | Tentative phytochemical | Formula | Theoretical MW | Reference ion | Average Peak Area (from Most Common Adduct) |
| --- | --- | --- | --- | --- | --- |
| 14 | Asparagine | C_4_H_8_N_2_O_3_ | 132.05349 | [M+H]^+^ | 2.48E+08 |
| 15 | Acetylcholine | C_7_H_15_NO_2_ | 145.11028 | [M+H]^+^ | 2.10E+08 |
| 16 | DL-Stachydrine | C_7_H_13_NO_2_ | 143.09463 | [M+H]^+^ | 1.88E+08 |
| 17 | D-(-)-Quinic acid | C_7_H_12_O_6_ | 192.06339 | [M+H]^+^ | 1.77E+08 |
| 18 | D-(+)-Pyroglutamic Acid | C_5_H_7_NO_3_ | 129.04259 | [M+H]^+^ | 1.73E+08 |
| 19 | Nicotinic acid | C_6_H_5_NO_2_ | 123.03203 | [M+H]^+^ | 1.70E+08 |
| 20 | L-Threonine | C_4_H_9_NO_3_ | 119.05824 | [M+H]^+^ | 1.65E+08 |
| 21 | Muramic acid | C_9_H_17_NO_7_ | 251.1005 | [M+H]^+^ | 1.39E+08 |
| 22 | Acetyl-L-carnitine | C_9_H_17_NO_4_ | 203.11576 | [M+H]^+^ | 1.12E+08 |
| 23 | N-Acetylornithine | C_7_H_14_N_2_O_3_ | 174.10044 | [M+H]+ | 8.37E+07 |
| 24 | 3,8,9-trihydroxy-10-propyl-3,4,5,8,9,10-hexahydro-2H-oxecin-2-one | C_12_H_20_O_5_ | 244.1311 | [M-H]^-^ | 9.29E+07 |
| 25 | 4-Hydroxybenzaldehyde | C_7_H_6_O_2_ | 122.0368 | [M+H]^+^ | 6.93E+07 |

**Table S5 (Continued**). Fraction 2

| No. | Tentative phytochemical | Formula | Theoretical MW | Reference ion | Average Peak Area (from Most Common Adduct) |
| --- | --- | --- | --- | --- | --- |
| 26 | N-Acetylneuraminic acid | C_11_H_19_NO_9_ | 309.1060 | [M+H]^+^ | 6.56E+07 |
| 27 | (-)-Caryophyllene oxide | C_15_H_24_O | 220.1827 | [M+H]^+^ | 5.17E+07 |
| 28 | Apocynin | C_9_H_10_O_3_ | 166.0630 | [M+H]^+^ | 4.11E+07 |
| 29 | Salsolinol | C_10_H_13_NO_2_ | 179.0946 | [M+H]^+^ | 3.72E+07 |
| 30 | L-Phenylalanine | C_9_H_11_NO_2_ | 165.0790 | [M+H]^+^ | 3.52E+07 |
| 31 | Sinapinic acid | C_11_H_12_O_5_ | 224.0685 | [M-H]^-^ | 2.25E+07 |
| 32 | 4-Hydroxy-3-(3-methyl-2-buten-1-yl) phenyl 6-O-[(2R,3R,4R)-3,4-dihydroxy-4-(hydroxymethyl)tetrahydro-2-furanyl]-beta-D-glucopyranoside | C_22_H_32_O_11_ | 472.1945 | [M-H]^-^ | 1.99E+07 |
| 33 | 5-O-Caffeoylquinic acid (Chlorogenic acid isomer) | C_16_H_18_O_9_ | 354.0951 | [M-H]^-^ | 1.49E+07 |
| 34 | 3-[3-(beta-D-Glucopyranosyloxy)-2-hydroxyphenyl] propanoic acid | C_15_H_20_O_9_ | 344.1107 | [M-H]^-^ | 1.07E+07 |

**Table S5 (Continued**). Fraction 2

| No. | Tentative phytochemical | Formula | Theoretical MW | Reference ion | Average Peak Area (from Most Common Adduct) |
| --- | --- | --- | --- | --- | --- |
| 35 | Coumarin | C_9_H_6_O_2_ | 146.0368 | [M+H]^+^ | 1.84E+07 |
| 36 | 4-Coumaric acid | C_9_H_8_O_3_ | 164.0473 | [M+H]^+^ | 1.43E+07 |
| 37 | L-Tyrosine | C_9_H_11_NO_3_ | 181.0739 | [M+H]^+^ | 1.29E+07 |
| 38 | α-Aspartylphenylalanine | C_13_H_16_N_2_O_5_ | 280.1059 | [M+H]^+^ | 1.10E+07 |
| 39 | 3-[3-(beta-D-Glucopyranosyloxy)-2-methoxyphenyl] propanoic acid | C_16_H_22_O_9_ | 358.1264 | [M-H]^-^ | 5.99E+06 |
| 40 | 4-O-Caffeoylquinic acid (Chlorogenic acid isomer) | C_16_H_18_O_9_ | 354.0951 | [M-H]^-^ | 3.81E+06 |
| 41 | 4-(beta-D-Glucopyranosyloxy)-2-methylenebutanoic acid | C_11_H_18_O_8_ | 278.1002 | [M-H]^-^ | 2.91E+06 |
| 42 | Gentisic acid 5-O-β-D-glucoside | C_13_H_16_O_9_ | 316.0794 | [M-H]^-^ | 2.77E+06 |
| 43 | D-(-)-Salicin | C_13_H_18_O_7_ | 286.1053 | [M-H]^-^ | 1.52E+06 |

**Table S6** LC–HRMS analysis of tentative phytochemical contents of F3 fractionated of WF-MLCE (S2-WF).

| No. | Tentative phytochemical | Formula | Theoretical MW | Reference ion | Average Peak Area (from Most Common Adduct) |
| --- | --- | --- | --- | --- | --- |
| 1 | Trigonelline | C_7_H_7_NO_2_ | 137.0477 | [M+H]^+^ | 6.91E+09 |
| 2 | L-Norleucine | C_6_H_13_NO_2_ | 131.0946 | [M+H]^+^ | 2.43E+09 |
| 3 | Pipecolic acid | C_6_H_11_NO_2_ | 129.0790 | [M+H]^+^ | 2.12E+09 |
| 4 | 3,8,9-trihydroxy-10-propyl-3,4,5,8,9,10-hexahydro-2H-oxecin-2-one (isomer 1) | C_12_H_20_O_5_ | 244.1311 | [M-H]^-^ | 1.71E+09 |
| 5 | Proline | C_5_H_9_NO_2_ | 115.0633 | [M+H]^+^ | 1.56E+09 |
| 6 | Gentisic acid 5-O-β-D-glucoside (isomer 1) | C_13_H_16_O_9_ | 316.0794 | [M-H]^-^ | 1.06E+09 |
| 7 | 1-deoxymannojirimycin or 1-deoxynorijimycin | C_6_H_13_NO_4_ | 163.0845 | [M+H]^+^ | 9.21E+08 |
| 8 | 4-Guanidinobutyric acid | C_5_H_11_N_3_O_2_ | 145.0851 | [M+H]^+^ | 8.48E+08 |
| 9 | L-Valine | C_5_H_11_NO_2_ | 117.0790 | [M+H]^+^ | 8.44E+08 |
| 10 | 3,8,9-trihydroxy-10-propyl-3,4,5,8,9,10-hexahydro-2H-oxecin-2-one (isomer 2) | C_12_H_20_O_5_ | 244.1311 | [M-H]^-^ | 6.88E+08 |
| 11 | Gentisic acid 5-O-β-D-glucoside (isomer 2) | C_13_H_16_O_9_ | 316.0794 | [M-H]^-^ | 4.93E+08 |

**Table S6 (Continued**). F3

| No. | Tentative phytochemical | Formula | Theoretical MW | Reference ion | Average Peak Area (from Most Common Adduct) |
| --- | --- | --- | --- | --- | --- |
| 12 | Nicotinic acid | C_6_H_5_NO_2_ | 123.0320 | [M+H]^+^ | 4.57E+08 |
| 13 | 3-[3-(beta-D-Glucopyranosyloxy)-2-hydroxyphenyl] propanoic acid (isomer 1) | C_15_H_20_O_9_ | 344.1107 | [M-H]^-^ | 3.85E+08 |
| 14 | Tropine | C_8_H_15_NO | 141.1154 | [M+H]^+^ | 3.53E+08 |
| 15 | 4-Hydroxybenzaldehyde | C_7_H_6_O_2_ | 122.0368 | [M+H]^+^ | 3.42E+08 |
| 16 | DL-Stachydrine | C_7_H_13_NO_2_ | 143.0946 | [M+H]^+^ | 2.85E+08 |
| 17 | Prolylleucine | C_11_H_20_N_2_O_3_ | 228.1474 | [M+H]^+^ | 2.82E+08 |
| 18 | Nicotinic acid | C_6_H_5_NO_2_ | 123.0320 | [M+H]^+^ | 2.51E+08 |
| 19 | Pantothenic acid | C_9_H_17_NO_5_ | 219.1107 | [M-H]^-^ | 2.33E+08 |
| 20 | 5-hydroxy-4-methoxy-5,6-dihydro-2H-pyran-2-one | C_6_H_8_O_4_ | 144.0423 | [M+H]^+^ | 1.85E+08 |
| 21 | DL-Carnitine | C_7_H_15_NO_3_ | 161.1052 | [M+H]^+^ | 1.12E+08 |
| 22 | 4-(4-hydroxy-2,6,6-trimethyl-3-([(2R,3R,4S,5S,6R)-3,4,5-trihydroxy-6-(hydroxymethyl)oxan-2-yl]oxy) cyclohex-1-en-1-yl) butan-2-one (isomer 1) | C_19_H_32_O_8_ | 388.2097 | [M+H]^+^ | 1.03E+08 |

**Table S6 (Continued**). F3

| No. | Tentative phytochemical | Formula | Theoretical MW | Reference ion | Average Peak Area (from Most Common Adduct) |
| --- | --- | --- | --- | --- | --- |
| 23 | Threonine | C_4_ H_9_ N O_3_ | 119.0582 | [M+H]^+^ | 7.46E+07 |
| 24 | N-Acetylornithine | C_7_ H_14_ N_2_ O_3_ | 174.1004 | [M+H]^+^ | 6.43E+07 |
| 25 | 3-[3-(beta-D-Glucopyranosyloxy)-2-hydroxyphenyl] propanoic acid (isomer 2) | C_15_ H_20_ O_9_ | 344.1107 | [M-H]^-^ | 5.62E+07 |
| 26 | (-)-Caryophyllene oxide (isomer 1) | C_15_H_24_O | 220.1827 | [M+H]^+^ | 4.70E+07 |
| 27 | Gentisic acid 5-O-β-D-glucoside (isomer 3) | C_13_H_16_O_9_ | 316.0794 | [M-H]^-^ | 4.61E+07 |
| 28 | (-)-Caryophyllene oxide (isomer 2) | C_15_H_24_O | 220.1827 | [M+H]^+^ | 3.98E+07 |
| 29 | N-Acetyl-DL-tryptophan | C_13_H_14_N_2_O_3_ | 246.1004 | [M-H]^-^ | 3.84E+07 |
| 30 | 3-[3-(beta-D-Glucopyranosyloxy)-2-methoxyphenyl] propanoic acid (isomer 3) | C_16_H_22_O_9_ | 358.1264 | [M-H]^-^ | 3.75E+07 |
| 31 | Gentisic acid 5-O-β-D-glucoside (isomer 4) | C_13_H_16_O_9_ | 316.0794 | [M-H]^-^ | 3.21E+07 |
| 32 | 2-Hexyl-3-methylenesuccinic acid | C_11_H_18_O_4_ | 214.1205 | [M+H]^+^ | 3.06E+07 |
| 33 | Sinapinic acid | C_11_H_12_O_5_ | 224.0685 | [M+H]^+^ | 2.88E+07 |

**Table S6 (Continued**). F3

| No. | Tentative phytochemical | Formula | Theoretical MW | Reference ion | Average Peak Area (from Most Common Adduct) |
| --- | --- | --- | --- | --- | --- |
| 34 | 4-(4-hydroxy-2,6,6-trimethyl-3-([(2R,3R,4S,5S,6R)-3,4,5-trihydroxy-6-(hydroxymethyl) oxan-2-yl] oxy) cyclohex-1-en-1-yl) butan-2-one (isomer 2) | C_19_H_32_O_8_ | 388.2097 | [M+H]^+^ | 2.67E+07 |
| 35 | 3-[3-(beta-D-Glucopyranosyloxy)-2-hydroxyphenyl] propanoic acid (isomer 4) | C_15_H_20_O_9_ | 344.1107 | [M-H]^-^ | 2.57E+07 |
| 36 | NP-008952 | C_12_H_20_O_4_ | 228.1362 | [M+H]^+^ | 2.26E+07 |
| 37 | Coumarin | C_9_H_6_O_2_ | 146.0368 | [M+H]^+^ | 2.01E+07 |
| 38 | 4'-Methoxyacetophenone | C_9_H_10_O_2_ | 150.0681 | [M+H]^+^ | 1.95E+07 |
| 39 | Curcolonol | C_15_H_20_O_4_ | 264.1362 | [M+H]^+^ | 1.84E+07 |
| 40 | 7-Hydroxycoumarine | C_9_H_6_O_3_ | 162.0317 | [M+H]^+^ | 1.74E+07 |
| 41 | Chlorogenic acid | C_16_H_18_O_9_ | 354.0951 | [M+H]^+^ | 1.58E+07 |
| 42 | 4-Coumaric acid | C_9_H_8_O_3_ | 164.0473 | [M+H]^+^ | 1.46E+07 |
| 43 | dihydrophaseic acid | C_15_H_22_O_5_ | 282.1467 | [M-H]^-^ | 1.31E+07 |

**Table S6 (Continued**). F3

| No. | Tentative phytochemical | Formula | Theoretical MW | Reference ion | Average Peak Area (from Most Common Adduct) |
| --- | --- | --- | --- | --- | --- |
| 44 | ((1S,5R,9R,13R)-1,5,9-trimethyl-11,14,15,16-tetraoxatetracyclo [10.3.1.04^,13^.0^8,13^] hexadecan-10-one or epi-Artemisinin | C_15_H_22_O_5_ | 282.1467 | [M+H]^+^ | 1.14E+07 |
| 45 | Thapsic acid | C_16_H_30_O_4_ | 286.2144 | [M+H]^+^ | 1.05E+07 |
| 46 | Jasmone | C_11_H_16_O | 164.1201 | [M+H]^+^ | 1.04E+07 |
| 47 | (1aR,1bR,2R,3R,7R,7aS)-1b,2-dimethyl-7a-(prop-1-en-2-yl)-1aH,1bH,2H,3H,4H,5H,7H,7aH-naphtho[1,2-b] oxirene-3,7-diol | C_15_H_22_O_3_ | 250.1569 | [M+H]^+^ | 9.87E+06 |
| 48 | L-Tyrosine | C_9_H_11_NO_3_ | 181.0739 | [M+H]^+^ | 9.82E+06 |
| 49 | 4-oxododecanedioic acid | C_12_H_20_O_5_ | 244.1311 | [M+H]^+^ | 9.35E+06 |
| 50 | 2-Hydroxy-2-methyl-3-buten-1-yl beta-D-glucopyranoside | C_11_H_20_O_7_ | 264.1209 | [M-H]^-^ | 8.70E+06 |
| 51 | Verrucarol | C_15_H_22_O_4_ | 266.1518 | [M+H]^+^ | 8.52E+06 |
| 52 | 5-(6-hydroxy-6-methyloctyl)-2,5-dihydrofuran-2-one | C_13_H_22_O_3_ | 226.1569 | [M+H]^+^ | 8.07E+06 |

**Table S6 (Continued**). F3

| No. | Tentative phytochemical | Formula | Theoretical MW | Reference ion | Average Peak Area (from Most Common Adduct) |
| --- | --- | --- | --- | --- | --- |
| 53 | Sinapinic acid | C_11_H_12_O_5_ | 224.0685 | [M-H]^-^ | 7.76E+06 |
| 54 | 6-Pentyl-2H-pyran-2-one | C_10_H_14_O_2_ | 166.0994 | [M+H]^+^ | 7.76E+06 |
| 55 | 12-oxo Phytodienoic Acid | C_18_H_28_O_3_ | 292.2038 | [M+H]^+^ | 7.66E+06 |
| 56 | (1r,3R,4s,5S)-4-([(2E)-3-(3,4-dihydroxyphenyl) prop-2-enoyl] oxy)-1,3,5-trihydroxycyclohexane-1-carboxylic acid | C_16_H_18_O_9_ | 354.0951 | [M+H]^+^ | 7.24E+06 |
| 57 | (1S,3R,4S,5R)-3,5-bis(([(2E)-3-(3,4-dihydroxyphenyl) prop-2-enoyl]oxy))-1,4-dihydroxycyclohexane-1-carboxylic acid | C_25_H_24_O_12_ | 516.1268 | [M+H]^+^ | 6.75E+06 |
| 58 | 4-O-Caffeoylquinic acid (Chlorogenic acid isomer) | C_16_H_18_O_9_ | 354.0951 | [M-H]^-^ | 6.64E+06 |
| 59 | Coumarin | C_9_H_6_O_2_ | 146.0368 | [M+H]^+^ | 6.58E+06 |

**Table S6 (Continued**). F3

| No. | Tentative phytochemical | Formula | Theoretical MW | Reference ion | Average Peak Area (from Most Common Adduct) |
| --- | --- | --- | --- | --- | --- |
| 60 | (4S)-4-hydroxy-3,5,5-trimethyl-4-[(1E)-3-([(2R,3R,4S,5S,6R)-3,4,5-trihydroxy-6-(hydroxymethyl) oxan-2-yl] oxy) but-1-en-1-yl] cyclohex-2-en-1-one | C_19_H_30_O_8_ | 386.1941 | [M+H]^+^ | 5.83E+06 |
| 61 | alpha-D-Glucopyranosyl 2-O-(2-methylbutanoyl)-alpha-D-glucopyranoside | C_17_H_30_O_12_ | 426.1737 | [M-H]^-^ | 5.70E+06 |
| 62 | Syringic acid | C_9_H_10_O_5_ | 198.0528 | [M-H]^-^ | 4.72E+06 |
| 63 | D-(-)-Salicin | C_13_H_18_O_7_ | 286.1053 | [M-H]^-^ | 4.53E+06 |

**Table S7** LC–HRMS analysis of tentative phytochemical contents of F4 fractionated of WF-MLCE (S2-WF).

| No. | Tentative phytochemical | Formula | Theoretical MW | Reference ion | Average Peak Area (from Most Common Adduct) |
| --- | --- | --- | --- | --- | --- |
| 1 | *trans*-5-O-Caffeoylquinic acid (Chlorogenic acid isomer) | C_16_H_18_O_9_ | 354.0951 | [M+H]^+^ | 6.80E+09 |
| 2 | *cis*-3-O-Caffeoylquinic acid (Chlorogenic acid isomer) | C_16_H_18_O_9_ | 354.0951 | [M-H]^-^ | 5.57E+09 |
| 3 | Gentisic acid 5-O-β-D-glucoside (isomer 1) | C_13_H_16_O_9_ | 316.0794 | [M-H]^-^ | 5.39E+09 |
| 4 | *cis*-4-O-Caffeoylquinic acid (Chlorogenic acid isomer) | C_16_H_18_O_9_ | 354.0951 | [M-H]^-^ | 4.88E+09 |
| 5 | Trigonelline | C_7_H_7_NO_2_ | 137.0477 | [M+H]^+^ | 4.03E+09 |
| 6 | *cis*-5-O-Caffeoylquinic acid (Chlorogenic acid isomer) | C_16_H_18_O_9_ | 354.0951 | [M-H]^-^ | 2.09E+09 |
| 7 | Gentisic acid 5-O-β-D-glucoside (isomer 2) | C_13_H_16_O_9_ | 316.0794 | [M-H]^-^ | 1.07E+09 |
| 8 | Proline | C_5_H_9_NO_2_ | 122.0368 | [M+H]^+^ | 1.03E+09 |
| 9 | 1-deoxymannojirimycin | C_6_H_13_NO_4_ | 115.0633 | [M+H]^+^ | 1.00E+09 |
| 10 | 4-Hydroxybenzaldehyde | C_7_H_6_O_2_ | 163.0845 | [M+H]^+^ | 9.76E+08 |
| 11 | 3-([(2R,3S,4S,5R,6S)-6-([2-(3,4-dihydroxyphenyl)-5,7-dihydroxy-4-oxo-4H-chromen-3-yl] ox)-3,4,5-trihydroxyoxan-2-yl] methoxy)-3-oxopropanoic acid | C_24_H_22_O_15_ | 550.0959 | [M+H]^+^ | 8.33E+08 |

**Table S7 (Continued**). F4

| No. | Tentative phytochemical | Formula | Theoretical MW | Reference ion | Average Peak Area (from Most Common Adduct) |
| --- | --- | --- | --- | --- | --- |
| 12 | 4-Guanidinobutyric acid | C_5_H_11_N_3_O_2_ | 135.0545 | [M+H]^+^ | 7.07E+08 |
| 13 | Uridine | C_9_H_12_N_2_O_6_ | 316.0794 | [M-H]^-^ | 6.97E+08 |
| 14 | Adenine | C_5_H_5_N_5_ | 145.0851 | [M+H]^+^ | 6.80E+08 |
| 15 | Gentisic acid 5-O-β-D-glucoside (isomer 3) | C_13_H_16_O_9_ | 244.0695 | [M-H]^-^ | 6.03E+08 |
| 16 | *trans*-4-O-Caffeoylquinic acid (Chlorogenic acid isomer) | C_16_H_18_O_9_ | 354.0951 | [M-H]^-^ | 5.66E+08 |
| 17 | 7-Hydroxycoumarine | C_9_H_6_O_3_ | 162.0317 | [M+H]^+^ | 4.37E+08 |
| 18 | *trans*-3-O-Caffeoylquinic acid (Chlorogenic acid isomer) | C_16_H_18_O_9_ | 354.0951 | [M-H]^-^ | 4.22E+08 |
| 19 | 12-oxo Phytodienoic Acid | C_18_H_28_O_3_ | 292.2038 | [M+H]^+^ | 4.19E+08 |
| 20 | Scopoletin | C_10_H_8_O_4_ | 192.0423 | [M+H]^+^ | 3.25E+08 |
| 21 | Caffeic acid | C_9_H_8_O_4_ | 180.0423 | [M+H]^+^ | 2.93E+08 |
| 22 | Esculetin | C_9_H_6_O_4_ | 178.0266 | [M+H]^+^ | 2.48E+08 |

**Table S7 (Continued**). F4

| No. | Tentative phytochemical | Formula | Theoretical MW | Reference ion | Average Peak Area (from Most Common Adduct) |
| --- | --- | --- | --- | --- | --- |
| 23 | 4-(4-hydroxy-2,6,6-trimethyl-3-([(2R,3R,4S,5S,6R)-3,4,5-trihydroxy-6-(hydroxymethyl) oxan-2-yl]oxy) cyclohex-1-en-1-yl) butan-2-one | C_19_H_32_O_8_ | 164.0473 | [M+H]^+^ | 2.27E+08 |
| 24 | 2-Hydroxycinnamic acid | C_9_H_8_O_3_ | 354.0951 | [M+H]^+^ | 2.01E+08 |
| 25 | (1S,3R,4R,5R)-1,3,4-trihydroxy-5-([(2E)-3-(4-hydroxy-3-methoxyphenyl) prop-2-enoyl]oxy) cyclohexane-1-carboxylic acid | C_17_H_20_O_9_ | 368.1107 | [M-H]^-^ | 1.85E+08 |
| 26 | (1r,3R,4s,5S)-4-([(2E)-3-(3,4-dihydroxyphenyl) prop-2-enoyl]oxy)-1,3,5-trihydroxycyclohexane-1-carboxylic acid | C_16_H_18_O_9_ | 388.2097 | [M+H]^+^ | 1.82E+08 |
| 27 | 5-hydroxy-4-methoxy-5,6-dihydro-2H-pyran-2-one | C_6_H_8_O_4_ | 144.0423 | [M+H]^+^ | 1.43E+08 |
| 28 | Nicotinic acid | C_6_H_5_NO_2_ | 123.0320 | [M+H]^+^ | 1.37E+08 |
| 29 | Dodecanedioic acid | C_12_H_22_O_4_ | 230.1518 | [M-H]^-^ | 1.27E+08 |
| 30 | Esculin | C_15_H_16_O_9_ | 340.0794 | [M-H]^-^ | 1.26E+08 |

**Table S7 (Continued**). F4

| No. | Tentative phytochemical | Formula | Theoretical MW | Reference ion | Average Peak Area (from Most Common Adduct) |
| --- | --- | --- | --- | --- | --- |
| 31 | Apocynin | C_9_H_10_O_3_ | 166.0630 | [M+H]^+^ | 1.08E+08 |
| 32 | 2-Hydroxyphenylalanine | C_9_H_11_NO_3_ | 181.0739 | [M+H]^+^ | 1.03E+08 |
| 33 | Coumarin | C_9_H_6_O_2_ | 146.0368 | [M+H]^+^ | 1.01E+08 |
| 34 | 2-Methoxybenzaldehyde | C_8_H_8_O_2_ | 368.1107 | [M+H]^+^ | 8.52E+07 |
| 35 | (4S)-4-hydroxy-3,5,5-trimethyl-4-[(1E)-3-([(2R,3R,4S,5S,6R)-3,4,5-trihydroxy-6-(hydroxymethyl) oxan-2-yl]oxy)but-1-en-1-yl]cyclohex-2-en-1-one | C_19_H_30_O_8_ | 136.0524 | [M+H]^+^ | 8.51E+07 |
| 36 | 2-(4-Hydroxyphenyl) ethyl 6-O-[(2R,3R,4R)-3,4-dihydroxy-4-(hydroxymethyl) tetrahydro-2-furanyl]-beta-D-glucopyranoside | C_19_H_28_O_11_ | 204.0899 | [M-H]^-^ | 8.25E+07 |
| 37 | (1S,3R,4R,5R)-1,3,4-trihydroxy-5-([(2E)-3-(4-hydroxy-3-methoxyphenyl) prop-2-enoyl] oxy}cyclohexane-1-carboxylic acid | C_17_H_20_O_9_ | 386.1941 | [M+H]^+^ | 7.58E+07 |

**Table S7 (Continued**). F4

| No. | Tentative phytochemical | Formula | Theoretical MW | Reference ion | Average Peak Area (from Most Common Adduct) |
| --- | --- | --- | --- | --- | --- |
| 38 | DL-Tryptophan | C_11_H_12_N_2_O_2_ | 432.1632 | [M-H]^-^ | 7.34E+07 |
| 39 | 5,7-dihydroxy-2-(3-hydroxy-4-([(2S,3R,4S,5S,6R)-3,4,5-trihydroxy-6-(hydroxymethyl) oxan-2-yl]oxy) phenyl)-4H-chromen-4-one | C_21_H_20_O_11_ | 448.1006 | [M+H]^+^ | 7.23E+07 |
| 40 | 2,3-Dihydroxybenzoic acid | C_7_H_6_O_4_ | 902.2692 | [M+H]^+^ | 6.20E+07 |
| 41 | (-)-Caryophyllene oxide | C_15_H_24_O | 150.1045 | [M+H]^+^ | 5.98E+07 |
| 42 | L-(-)-Carvone | C_10_H_14_O | 154.0266 | [M+H]^+^ | 5.95E+07 |
| 43 | 7-([(2S,3R,4S,5S,6R)-4,5-dihydroxy-6-(hydroxymethyl)-3-([(2S,3R,4R,5R,6S)-3,4,5-trihydroxy-6-methyloxan-2-yl]oxy)oxan-2-yl]oxy)-5-hydroxy-2-(4-hydroxyphenyl)-3-([(2S,3R,4S,5S,6R)-3,4,5-trihydroxy-6-(([(2R,3R,4R,5R,6S)-3,4,5-trihydroxy-6-methyloxan-2-yl]oxy)methyl)oxan-2-yl]oxy)-4H-chromen-4-one | C_39_H_50_O_24_ | 154.0266 | [M+H]^+^ | 6.82E+07 |

**Table S7 (Continued**). F4

| No. | Tentative phytochemical | Formula | Theoretical MW | Reference ion | Average Peak Area (from Most Common Adduct) |
| --- | --- | --- | --- | --- | --- |
| 44 | 3-([(2R,3S,4S,5R,6S)-6-([2-(3,4-dihydroxyphenyl)-5,7-dihydroxy-4-oxo-4H-chromen-3-yl]oxy)-3,4,5-trihydroxyoxan-2-yl]methoxy)-3-oxopropanoic acid | C_24_H_22_O_15_ | 220.1827 | [M+H]^+^ | 5.85E+07 |
| 45 | 2,4-Dihydroxybenzoic acid | C_7_H_6_O_4_ | 550.0959 | [M+H]^+^ | 5.62E+07 |
| 46 | 5-O-(4-coumaroyl)-D-quinic acid (isomer 1) | C_16_H_18_O_8_ | 358.1264 | [M-H]^-^ | 5.07E+07 |
| 47 | 5-O-(4-coumaroyl)-D-quinic acid (isomer 2) | C_16_H_18_O_8_ | 338.1002 | [M-H]^-^ | 4.88E+07 |
| 48 | 3-[3-(beta-D-Glucopyranosyloxy)-2-methoxyphenyl]propanoic acid | C_16_H_22_O_9_ | 338.1002 | [M-H]^-^ | 4.57E+07 |
| 49 | 3-[3-(beta-D-Glucopyranosyloxy)-2-hydroxyphenyl]propanoic acid | C_15_H_20_O_9_ | 344.1107 | [M-H]^-^ | 3.66E+07 |
| 50 | (1S,3R,4R,5R)-1,3,4-trihydroxy-5-([(2E)-3-(4-hydroxy-3-methoxyphenyl)prop-2-enoyl]oxy)cyclohexane-1-carboxylic acid | C_17_H_20_O_9_ | 368.1107 | [M-H]^-^ | 3.03E+07 |
| 51 | N-Acetyl-DL-tryptophan | C_13_H_14_N_2_O_3_ | 368.1107 | [M-H]^-^ | 2.60E+07 |

**Table S7 (Continued**). F4

| No. | Tentative phytochemical | Formula | Theoretical MW | Reference ion | Average Peak Area (from Most Common Adduct) |
| --- | --- | --- | --- | --- | --- |
| 52 | Riboflavin | C_17_H_20_N_4_O_6_ | 246.1004 | [M-H]^-^ | 2.59E+07 |
| 53 | 1,3,5-trihydroxy-4-([(2E)-3-(3-hydroxy-4-methoxyphenyl)prop-2-enoyl]oxy)cyclohexane-1-carboxylic acid | C_17_H_20_O_9_ | 286.1053 | [M-H]^-^ | 2.58E+07 |
| 54 | D-(-)-Salicin | C_13_H_18_O_7_ | 376.1383 | [M-H]^-^ | 2.31E+07 |
| 55 | Benzyl 6-O-beta-D-glucopyranosyl-beta-D-glucopyranoside | C_19_H_28_O_11_ | 432.1632 | [M-H]^-^ | 2.29E+07 |
| 56 | (±)-Abscisic acid | C_15_H_20_O_4_ | 264.1362 | [M-H]^-^ | 1.35E+07 |
| 57 | D-(-)-Salicin | C_13_H_18_O_7_ | 286.1053 | [M-H]^-^ | 1.27E+07 |
| 58 | 5-Hydroxytryptophan | C_11_H_12_N_2_O_3_ | 220.0848 | [M-H]^-^ | 9.43E+06 |
| 59 | 2-(4-Hydroxyphenyl)ethyl 6-O-[(2R,3R,4R)-3,4-dihydroxy-4-(hydroxymethyl)tetrahydro-2-furanyl]-beta-D-glucopyranoside | C_19_H_28_O_11_ | 432.1632 | [M-H]^-^ | 6.84E+06 |

**Table S7 (Continued**). F4

| No. | Tentative phytochemical | Formula | Theoretical MW | Reference ion | Average Peak Area (from Most Common Adduct) |
| --- | --- | --- | --- | --- | --- |
| 60 | 1,3,5-trihydroxy-4-([(2E)-3-(3-hydroxy-4-methoxyphenyl)prop-2-enoyl]oxy) cyclohexane-1-carboxylic acid | C_17_H_20_O_9_ | 368.1107 | [M-H]^-^ | 5.20E+06 |


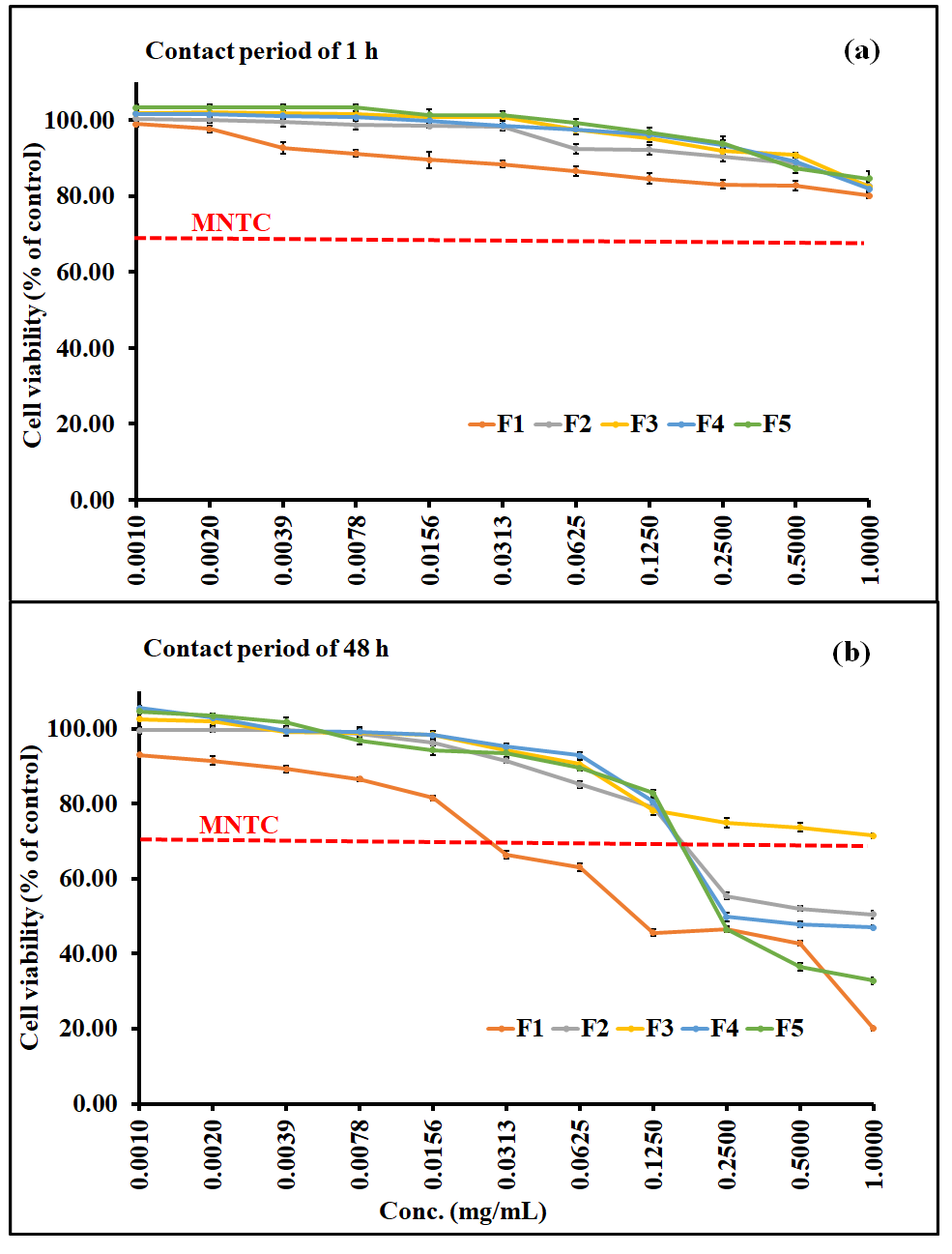


**Fig. S4** Cytotoxicity of the five fractions obtained from column chromatography of WF-MLCE, evaluated over Vero E6 cells by MTT assay. Results are expressed as % cell viability (mean ± SD). (a) examined with a contact period of 1 h, and (b) examined with a contact period of 48 h. MNTC-red dot line indicates the minimum non-toxicity concentration (MNTC) with 70% cell viability of individual crude extract.

**Table S8** Anti-SARS-CoV-2 efficacy of the five fractions (F1-F5) obtained from column chromatography of WF-MLCE.

| Fraction | Pre-entry study | | |  | Postinfection treatment study | | |
| --- | --- | --- | --- | --- | --- | --- | --- |
|  | Conc.  (mg/mL) | Anti-SARS-CoV-2 efficacy | |  | Conc.  (mg/mL) | Anti-SARS-CoV-2 efficacy | |
|  |  | Log Reduction | % Virucidal |  |  | Log Reduction | % Inhibition |
| F1 | 1.00 | 3.31 ± 0.22 | 99.95 ± 0.03 |  | 0.0156 | 2.44 ± 0.07 | 99.65 ± 0.07 |
|  | 0.50 | 3.19 ± 0.30 | 99.93 ± 0.04 |  | 0.0078 | 2.65 ± 0.27 | 99.74 ± 0.17 |
|  | 0.25 | 3.02 ± 0.27 | 99.90 ± 0.04 |  | 0.0039 | 2.65 ± 0.27 | 99.74 ± 0.17 |
| F2 | 1.00 | 2.69 ± 0.30 | 99.78 ± 0.11 |  | 0.0625 | 2.69 ± 0.30 | 99.78 ± 0.11 |
|  | 0.50 | 2.56 ± 0.22 | 99.73 ± 0.08 |  | 0.0313 | 2.69 ± 0.30 | 99.78 ± 0.11 |
|  | 0.25 | 2.44 ± 0.07 | 99.65 ± 0.07 |  | 0.0156 | 2.69 ± 0.30 | 99.78 ± 0.11 |
| F3 | 1.00 | 2.78 ± 0.18 | 99.82 ± 0.10 |  | 0.0625 | 2.77 ± 0.18 | 99.82 ± 0.10 |
|  | 0.50 | 2.52 ± 0.14 | 99.71 ± 0.05 |  | 0.0313 | 2.60 ± 0.21 | 99.75 ± 0.08 |
|  | 0.25 | 2.10 ± 0.22 | 99.21 ± 0.26 |  | 0.0156 | 2.52 ± 0.14 | 99.71 ± 0.05 |
| F4 | 1.00 | 2.81 ± 0.22 | 99.83 ± 0.01 |  | 0.1250 | 2.56 ± 0.30 | 99.70 ± 0.15 |
|  | 0.50 | 2.44 ± 0.07 | 99.65 ± 0.07 |  | 0.0625 | 2.56 ± 0.30 | 99.70 ± 0.15 |
|  | 0.25 | 2.27 ± 0.30 | 99.36 ± 0.56 |  | 0.0313 | 2.52 ± 0.22 | 99.69 ± 0.13 |
| F5 | 1.00 | 3.73 ± 0.38 | 99.97 ± 0.03 |  | 0.1250 | 1.94 ± 0.07 | 98.88 ± 0.23 |
|  | 0.50 | 3.15 ± 0.38 | 99.92 ± 0.04 |  | 0.0625 | 2.35 ± 0.38 | 99.46 ± 0.38 |
|  | 0.25 | 2.77 ± 0.07 | 99.84 ± 0.03 |  | 0.0313 | 2.81 ± 0.07 | 99.85 ± 0.00 |

Results are expressed as mean ± SD of quadruplicate experiments; Conc., tested concentration of fraction; Log reduction, the reduction of viral titer after experimental treatment in logarithmic function; % Inhibition or % Virucidal, % viral titer reduction by comparing with the initial viral load.

**Table S9** Polyphenol contents and antioxidant activities of the fractions obtained from column chromatography of WF-MLCE

| Fraction | TPC  (mg GAE/g) | TFC  (mg QE/g) | FRAP value | |  | DPPH^•^ scavenging  activity | |  | ABTS^•+^ scavenging activity | |
| --- | --- | --- | --- | --- | --- | --- | --- | --- | --- | --- |
|  |  |  | μmol TE/g | μmol Fe2^+^/g |  | μmol TE/g | EC_50_  (mg/mL) |  | μmol TE/g | EC_50_  (mg/mL) |
| F1 | 16.90^c^  ± 0.74 | 54.50^c^  ± 5.56 | 43.48^c^  ± 2.11 | 79.40^c^  ± 3.91 |  | 33.11^c^  ± 3.48 | 5.45^b^  ± 0.26 |  | 131.03^c^  ± 5.53 | 2.91^b^  ± 0.30 |
| F2 | 6.73 ^d^  ± 0.70 | 8.20^d^  ± 1.22 | 24.23^c^  ± 1.63 | 44.38^c^  ± 3.02 |  | 12.24 ^d^  ± 0.65 | 9.45^c^  ± 1.49 |  | 53.97^c^  ± 3.54 | 7.51^c^  ± 0.99 |
| F3 | 8.53^cd^  ± 0.78 | 11.28^d^  ± 1.23 | 39.81^c^  ± 3.62 | 72.79^c^  ± 6.70 |  | 22.29 ^cd^  ± 2.21 | 6.81^b^  ± 1.21 |  | 83.90^c^  ± 9.76 | 6.07^c^  ± 0.81 |
| F4 | 76.88^b^  ± 9.16 | 131.45^b^  ± 9.81 | 399.64^b^  ± 23.35 | 730.79^b^  ± 43.11 |  | 259.49 ^b^  ± 22.95 | 0.54^a^  ± 0.08 |  | 640.24^b^  ± 5.75 | 0.67^a^  ± 0.08 |
| F5 | 158.10^a^  ± 12.08 | 565.88^a^  ± 19.71 | 1269.38^a^  ± 179.24 | 2322.42^a^  ± 323.25 |  | 532.58 ^a^  ± 18.90 | 0.23^a^  ± 0.02 |  | 2475.63^a^  ± 182.40 | 0.13^a^  ± 0.03 |

Results are expressed as mean ± SD on dry weight basis in triplicate experiment. The values with significant differences (*p* ≤ 0.05) are indicated within the same column by different superscript letters. F1-F5, Fraction 1-5; TPC, total phenolic content; TFC, total flavonoid content; FRAP, ferric reducing antioxidant power assay; EC50, effective concentration that performed 50% antioxidant activity; GAE, gallic acid equivalents; QE, quercetin equivalents; and TE, trolox equivalents.
